# New records and an updated herpetofaunal list from Ba Vi National Park, Vietnam

**DOI:** 10.3897/BDJ.14.e178266

**Published:** 2026-05-19

**Authors:** Tuong Sy Dinh, Kanto Nishikawa, Vinh Quang Luu, Huy Quang Nguyen, Shinya Numata

**Affiliations:** 1 Graduate School of Urban Environmental Sciences, Tokyo Metropolitan University, Tokyo, Japan Graduate School of Urban Environmental Sciences, Tokyo Metropolitan University Tokyo Japan; 2 Graduate School of Human and Environmental Studies, Kyoto University, Yoshida-nihonmatsu-cho, Sakyo, Kyoto, Japan Graduate School of Human and Environmental Studies, Kyoto University, Yoshida-nihonmatsu-cho, Sakyo Kyoto Japan; 3 The Geopark Research Centre, Institute for Environment and Development (LESTARI), University Kebangsaan Malaysia, UKM Bangi, Selangor, Malaysia The Geopark Research Centre, Institute for Environment and Development (LESTARI), University Kebangsaan Malaysia, UKM Bangi Selangor Malaysia; 4 Graduate School of Global Environmental Studies, Kyoto University, Yoshida-hon-machi, Sakyo, Kyoto, Japan Graduate School of Global Environmental Studies, Kyoto University, Yoshida-hon-machi, Sakyo Kyoto Japan; 5 Department of Biology, Faculty of Science, Chulalongkorn University, Bangkok, Thailand Department of Biology, Faculty of Science, Chulalongkorn University Bangkok Thailand; 6 Vietnam National University of Forestry, Hanoi, Vietnam Vietnam National University of Forestry Hanoi Vietnam; 7 Ba Vi National Park, Hanoi, Vietnam Ba Vi National Park Hanoi Vietnam

**Keywords:** Amphibians, reptiles, species richness, taxonomy

## Abstract

**Background:**

The Ba Vi National Park is located 60 km west of Hanoi, the capital city, in south Phu Tho Province (formerly Hoa Binh Province), northern Vietnam. Despite its establishment in 1991, there are not many studies on its herpetofauna. The known number of amphibian and reptile species for the National Park prior to this study is a total 66 species. However, due to the scarcity of surveys, more species were expected.

**New information:**

Herein, we report new records of 16 amphibians and 12 reptiles from Ba Vi National Park. Our findings increase the total number of herpetofaunal species to 94, including now a total of 26 anurans, one gymnophionan, four chelonians, 18 saurians and 45 ophidians. The estimated species accumulation curve has not reached a plateau yet and thus indicates that species richness in the Park is very likely to increase in the future.

## Introduction

As part of one of the leading biodiversity hotspots in Indo-Burma (Smith and Matimele 2025), Vietnam is recognised as a country with exceptionally high levels of biodiversity and is considered a priority area for conservation due to rapid habitat loss and a large number of endemic species (Krzikowski et al. 2022, Stenger et al. 2023). However, biodiversity in Vietnam remains underestimated. Even for relatively well-studied terrestrial vertebrates, knowledge of species diversity and distribution is still incomplete. In order to better understand the historical formation of biodiversity in Indo-Burma and Southeast Asia and to develop effective conservation strategies, it is essential to clarify the true extent of biodiversity in Vietnam.

Ba Vi National Park (hereafter, BVNP) is located in northern Vietnam near the confluence of the Red River and the Black River, two major river systems recognised as important biogeographic features in Indochina (Bain and Hurley 2011, Abramov et al. 2013). Due to its proximity to Hanoi, the capital of Vietnam, BVNP is considered one of the relatively well-explored areas for vertebrate fauna. Nevertheless, knowledge of its biodiversity remains incomplete. Amphibians and reptiles are particularly suitable indicators for biodiversity assessments (Stuart et al. 2008, Jacob et al. 2024) because they exhibit high levels of species richness and endemism (Frost 2026, Uetz et al. 2026). Nguyen et al. (2009) reported nine species of amphibians representing five families and 43 species of reptiles representing 14 families from BVNP. Subsequently, 14 additional species (two amphibians and 12 reptiles) were recorded (Nguyen et al. 2011b, Chen et al. 2018, Luu et al. 2020, Orlov et al. (2020), Nguyen et al. 2021, Pham et al. 2025). Despite these efforts, the true diversity of amphibians and reptiles in BVNP remains uncertain.

In this study, we conducted a re-inventory of amphibians and reptiles in BVNP. The results provide an updated assessment of species diversity in a relatively well-explored protected area and allow us to evaluate how much biodiversity may still be underestimated. These findings contribute not only to local conservation planning in BVNP, but also to a broader understanding of biodiversity patterns in Vietnam and the Indo-Burma Region, particularly in protected areas located near rapidly developing urban centres.

## Materials and methods

**Study area**

Ba Vi National Park (BVNP) is located Ba Vi District of Hanoi City and Luong Son and Ky Son districts of Phu Tho Province (formerly Hoa Binh Province), with a total area of 10,814.6 ha (Luu et al. 2020). The habitat of BVNP is characterised by a typical subtropical forest at lower elevations, but by evergreen mixed forest of coniferous and broadleaf trees above 600 m. In terms of climatic conditions, BVNP is located in the subtropical climate region of northern Vietnam, with annual average rainfall, temperature and humidity of 900 mm, 21.8°C and 79%, respectively (Pham et al. 2025). Ba Vi is under the influence of a monsoon tropical climate with a cold winter and summer rains, with a dry period of ca. 3.0 months; the per-humid period extends from the beginning of April to the end of October (Eguchi et al. 2004).

**Field surveys**

**Figure 1.**
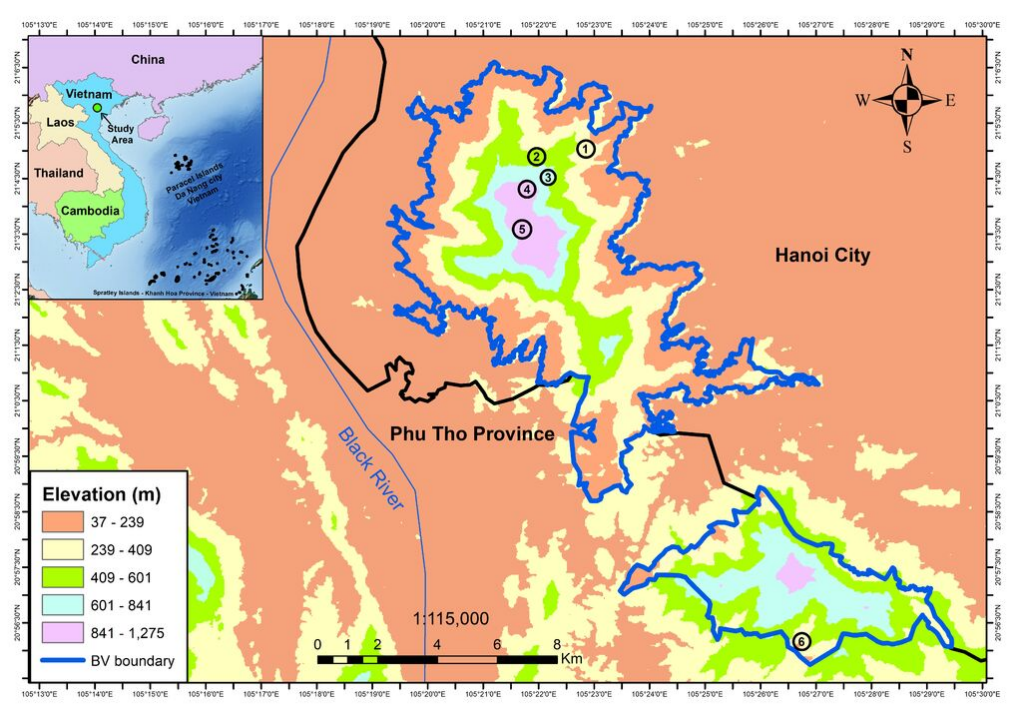
Topography map of BVNP. The numbers indicate sites: **1** Ngoc Hoa Cave; **2** Ngoc Hoa Stream; **3** the French camping area; **4** the ruins of the Old French Church; **5** the French Era Political Prison (ruins) and Thuong temple; **6** Thang Thien Waterfall – Phu Tho Province (formerly Hoa Binh Province). The 30 m resolution Shuttle Radar Topography Mission (SRTM) digital elevation data were extracted from Google Earth Engine (GEE) (NASA JPL 2013).

**Figure 2.**
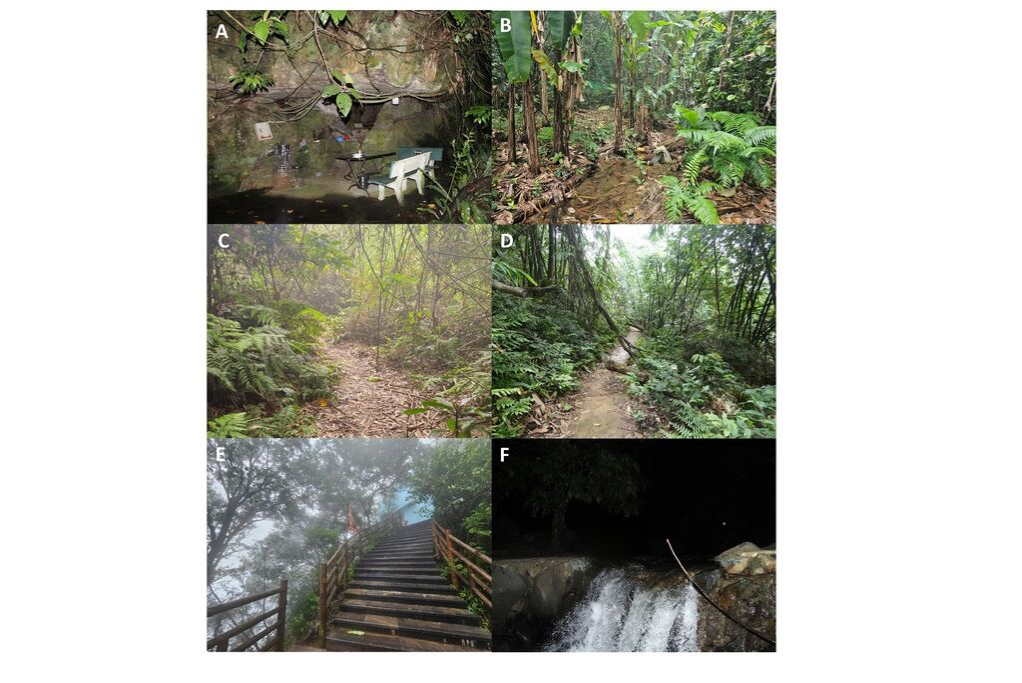
Surveyed habitats on BVNP. **A** Humid rock crevice habitat; **B** Dry streambed in secondary forest; **C** Moist secondary forest habitat along a forest trail; **D** Evergreen broad-leaved forest with bamboo; **E** Evergreen broadleaf forest along a tourist trail; **F** Rocky stream habitat with a natural waterfall in evergreen forest. Photographs by Dinh T.S. and Nguyen H.Q.

Field surveys were conducted in the BVNP from 6 to 15 June 2019 by Luu V.Q. and Lo O.A. in Ba Vi District; from 15 March to 21 April 2021 by Dinh T.S., Nguyen H.Q. and Nishikawa K.; from 1 July to 4 August 2024 in Ba Vi and Ky Son Districts by Dinh T.S. and Nguyen H.Q.; and from 11 June to 1 August 2025 by Dinh T.S., Nguyen Q.H., Nishikawa K. and Inoue K in Ba Vi and Ky Son Districts. Survey sites and collection of specimens were selected at elevations ranging from 200 to 1100 m a.s.l, took place after sunset between 19:00 h and 23:00 h mainly along forest trails, streams, puddles and old ruins from the French colonial era in six sites (Fig. 1 and Fig. 2). The coordinates (WGS 84) and elevations were determined by using a Garmin 78s device. Specimens were collected by hand (frog and lizard), by tools for snakes. After taking photographs in life, specimens were anaesthetised and euthanised in a closed vessel with a piece of cotton wool soaked in ethyl acetate (Simmons 2002), fixed in 10% formalin and later transferred to 70% ethanol for permanent storage. The specimens were subsequently deposited in the collection of the Vietnam National University of Forestry (VNUF) and Institute of Biology (IB) Hanoi, Vietnam.

**Species identification and morphological description**

Measurements were taken with a digital calliper Mitutoyo to the nearest 0.1 mm. For amphibians following Pham et al. (2020), Luong et al. (2021) and Le et al. (2021), abbreviations are as follows: SVL = Snout-vent length (from tip of snout to anterior margin of cloaca); HL = Head length (from posterior corner of mandible to tip of snout); HW = Maximum head width (across angles of jaws). For reptiles following Luu et al. (2020) and Le et al. (2021), abbreviations are as follows: SVL = Snout-vent length (from tip of snout to anterior margin of cloaca); TaL = Tail length (from posterior margin of cloaca to tip of tail); HL = Head length (from the tip of snout to back of mandible); HW = Maximum head width (across angles of jaws). For snake specimens, measurements were taken after preservation with a measuring tape. The number of ventral scales was counted according to Dowling (1951). The dorsal scale rows were given at one head length behind head, at mid-body and at one head length before vent, respectively. Scalation was counted by using a digital microscope (Terino–HD1600X–12MP).

**Species increment and rarefaction analyses**

Species increment (species accumulation by survey day) curves were constructed in Microsoft Excel, based on cumulative species records across survey days. Sampling completeness was further evaluated using sample-based rarefaction curves and incidence-based richness estimators implemented in R with the package vegan (Oksanen et al. 2022). Rarefaction curves were generated using the Mao Tau method and non-parametric estimators (Chao2, Jackknife 2 and Bootstrap) were calculated to estimate expected species richness for the study area.

**Use of Artificial Intelligence in manuscript preparation**

During manuscript preparation, ChatGPT (OpenAI) was used exclusively to improve English language quality, including grammar, sentence structure and clarity of expression. The AI tool was not involved in generating scientific content, data analysis, interpretation or drawing conclusions.

## Checklists

### An updated checklist of the herpetofauna of Ba Vi National Park

#### Bufonidae

3FBC7CA5-7362-5716-8E39-A8C5CE90CD65

#### Duttaphrynus melanostictus

(Schneider, 1799)

B102A511-1573-577F-84D7-9131C64732C3

##### Native status

Not threatened

##### Conservation status

LC (IUCN 2025)

##### Distribution

Vietnam (entire country: Nguyen et al. (2009)), Sri Lanka, India, Nepal, China, Myanmar, Laos, Thailand, Cambodia, Malaysia and the Philippines (Nguyen et al. 2009).

##### Notes

Previously recorded from BVNP by Nguyen et al. (2009), observed during the present surveys.

#### Dicroglossidae

132038D9-7947-5A59-B386-5B5F55B487D3

#### Fejervarya limnocharis

(Gravenhorst, 1829)

B4AA6863-A9E6-5CEA-B046-87173A47AB09

##### Native status

Not threatened

##### Conservation status

LC (IUCN 2025)

##### Distribution

Vietnam (entire country: Nguyen et al. (2009)), Afghanistan, Pakistan, India, Nepal, Sri Lanka, Bangladesh, China, Myanmar, Laos, Thailand, Cambodia, Malaysia, Singapore, Indonesia and the Philippines (Nguyen et al. 2009 with a correction).

##### Notes

Previously recorded from BVNP by Nguyen et al. (2009), observed during the present surveys.

#### Hoplobatrachus chinensis

(Osbeck, 1765)

640504A2-2C7A-5D0D-A8E3-728D0C223BA8

##### Native status

Not threatened

##### Conservation status

Not threatened

##### Distribution

Vietnam (entire country: Nguyen et al. (2009)), China, Taiwan, Myanmar, Laos, Thailand, Cambodia and Malaysia (Nguyen et al. 2009).

##### Notes

Previously recorded from BVNP by Nguyen et al. (2009), not observed during the present surveys.

#### Limnonectes bannaensis

Ye, Fei & Jiang, 2007

C63A4C51-05F1-5286-84DA-39FE7C9A58CC

##### Native status

Not threatened

##### Conservation status

LC (IUCN 2025)

##### Distribution

Vietnam (Cao Bang, Ba Vi: Nguyen et al. (2009), Quang Ninh, Phu Yen, Ha Giang, Ha Tinh, Lai Chau and Dien Bien Provinces) along the highlands to the Central Highlands, China, Laos, Myanmar and Thailand (Frost 2026).

##### Notes

Previously recorded from BVNP by Nguyen et al. (2009); observed during the present surveys.

#### Occidozyga martensii

(Peters, 1867)

4384E1EF-B040-55B7-AFC7-147AB26B766F

##### Materials

- **Type status:** Other material. **Occurrence:** catalogNumber: VNUF R.2024.88; individualCount: 1; sex: male; lifeStage: adult; occurrenceID: 5703C230-3EE2-52B6-AE1C-E949444E8271; **Taxon:** genus: Occidozyga; specificEpithet: *martensii*; **Location:** country: Vietnam; stateProvince: Phu Tho; municipality: Ky Son; locality: Ba Vi National Park; verbatimElevation: 173 m; verbatimCoordinates: 20 55.721N, 105 26.536E; verbatimCoordinateSystem: degrees decimal minutes; **Event:** eventDate: 29 July 2024; **Record Level:** institutionCode: VNUF
- **Type status:** Other material. **Occurrence:** catalogNumber: VNUF R.2024.92; individualCount: 1; sex: male; lifeStage: adult; occurrenceID: 06E87DC7-27F6-52A3-B5DA-840CB0CC83F1; **Taxon:** genus: Occidozyga; specificEpithet: *martensii*; **Location:** country: Vietnam; stateProvince: Phu Tho; municipality: Ky Son; locality: Ba Vi National Park; verbatimElevation: 173 m; verbatimCoordinates: 20 55.721N, 105 26.536E; verbatimCoordinateSystem: degrees decimal minutes; **Event:** eventDate: 29 July 2024; **Record Level:** institutionCode: VNUF
- **Type status:** Other material. **Occurrence:** catalogNumber: VNUF R.2024.90; individualCount: 1; sex: female; lifeStage: adult; occurrenceID: 9BFB71D1-CA4D-5E20-AC70-EA9EA43CFE76; **Taxon:** genus: Occidozyga; specificEpithet: *martensii*; **Location:** country: Vietnam; stateProvince: Phu Tho; municipality: Ky Son; locality: Ba Vi National Park; verbatimElevation: 173 m; verbatimCoordinates: 20 55.721N, 105 26.536E; verbatimCoordinateSystem: degrees decimal minutes; **Event:** eventDate: 29 July 2024; **Record Level:** institutionCode: VNUF

##### Native status

Not threatened

##### Conservation status

LC (IUCN 2025)

##### Distribution

In Vietnam, this species was previously known from Lao Cai Province in the north to Dong Nai and Ba Ria-Vung Tau Provinces in the south (Hecht et al. 2013). Elsewhere, this species has been reported from Malaysia, China, Laos, Thailand, andCambodia (Vassilieva et al. 2016). First record in BVNP.

##### Notes

The specimens were found at 22:30 h along puddles near the path. The surrounding habitat was a tourism area, within Thang Thien Waterfall ecotourism area. The relative temperature was 26°C and the humidity was 89%.

##### Diagnosis

**Figure 3.**
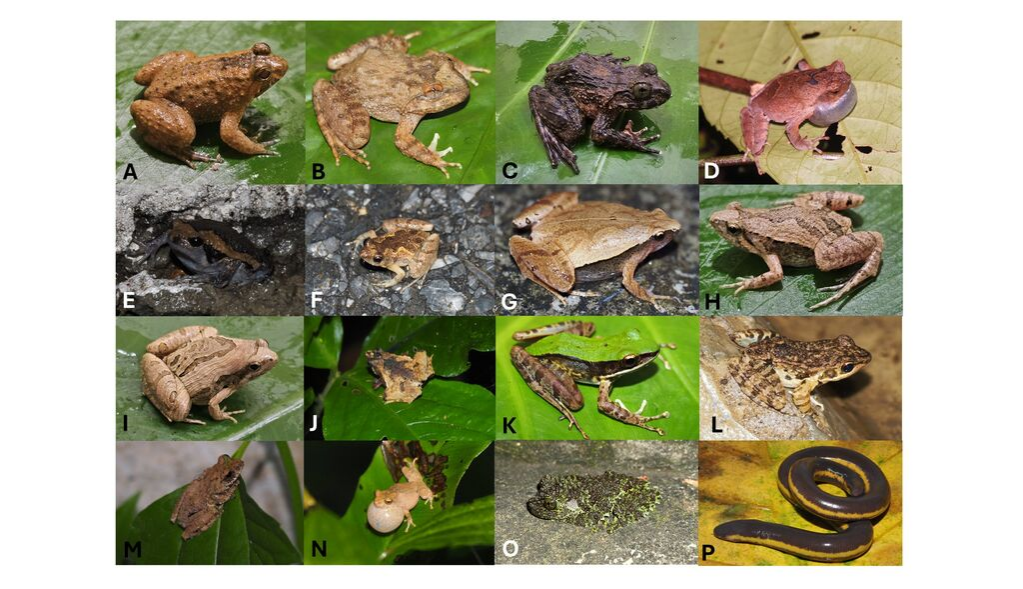
Sixteen newly-recorded amphibian species from BVNP. **A** *Occidozyga martensii*; **B** Quasipaa cf. spinosa; **C** *Quasipaa verrucospinosa*; **D** *Ophryophryne microstoma*; **E** *Kaloula pulchra*; **F** *Microhyla butleri*; **G** Microhyla cf. heymonsi; **H** *Microhyla mukhlesuri*; **I** *Microhyla pulchra*; **J** *Nanohyla marmorata*; **K** *Hylarana maosonensis*; **L** *Odorrana chloronota*; **M** *Kurixalus bisacculus*; **N** *Raorchestes parvulus*; **O** *Theloderma corticale*; **P** *Ichthyophis kohtaoensis*. Photographs by Dinh T.S. & K. Nishikawa.

Morphological characters of the specimens from BVNP agreed well with descriptions of Hecht et al. (2013) and Gawor et al. (2016): SVL 25.2 mm in two males and 32.2 mm in the female; head longer than wide (HL 8.3–8.8, HW 8.2–8.7 mm in males and HL 11.1 mm, HW 10.8 mm in the female), head rather flattened; with short triangular snout; tympanum indistinct; eyes bulging, orientated laterally, pupils diamond-shaped; vomerine teeth absent; tongue rounded posteriorly. Fore-limbs: short; finger free of webbing; males with nuptial pad on finger I. Hind-limbs: thigh shorter; toes fully webbed. Skin: dorsum with small, scattered tubercles; ventral skin smooth. Colouration in life: dorsal surface brown; head with a thin transverse dark line between eyes; broad brownish stripe on back arising at posterior corner of eyes; supratympanic fold black; one light vertical bar from eye to snout; limbs with transverse bars; ventral side of body cream with a dark marbled throat (Fig. 3)

#### Quasipaa cf. spinosa

(David, 1875)

C4401F85-779D-572C-A853-7571EBFA419E

##### Materials

- **Type status:** Other material. **Occurrence:** catalogNumber: IB A.2025.032; individualCount: 1; sex: male; lifeStage: juvenile; occurrenceID: 49B7D627-14B1-526C-87F7-68AA79BD3BDD; **Taxon:** genus: Quasipaa; specificEpithet: cf. *spinosa*; **Location:** country: Vietnam; stateProvince: Hanoi; municipality: Ba Vi; locality: Ba Vi National Park; verbatimElevation: 1000 m; verbatimCoordinates: 21 03.544N, 105 21.637E; verbatimCoordinateSystem: degrees decimal minutes; **Event:** eventDate: 15 June 2025; **Record Level:** institutionCode: IB

##### Native status

Not threatened

##### Conservation status

VU (IUCN 2025)

##### Distribution

In Vietnam, this species is known from Lao Cai to Hoa Binh Provinces in the north, southwards to Central Highlands - Gia Lai Province (Nguyen et al. 2009). Elsewhere, this species has been reported from China (Frost 2026). First record in BVNP.

##### Notes

The specimen was found on the ground at 22:00 h in the French Era Political Prison (ruins). The surrounding habitats were evergreen forest, streams and cliffs. The relative temperature was 25°C and humidity was 90%.

##### Diagnosis

Morphological characters of the specimens from BVNP agreed well with descriptions of Boulenger (1920): SVL 56.7 mm; head broader than long (HL 23.6 mm, HW 24.5 mm), much depressed; snout rounded; canthus rostralis very obtuse; loreal region very oblique, slightly concave; eyes bulging, orientated laterally, pupils diamond-shaped; tympanum indistinct; vomerine teeth present. Fore-limbs: Fingers feebly swollen at the tips, first longer than the second. Hind-limbs: moderately long; toes with the tips swollen into small discs, entirely webbed. Skin: dorsal surface of head and body granular, back with large oval warts intermixed with small tubercles; sides of belly covered by oval tubercles with dark spines; ventral surface smooth. Colouration in life: dorsal blackish brown above; lips with darker vertical bars; limbs with distinct black cross-bars; thighs brown with distinct black marblings. Lower parts whitish (Fig. 3).

#### Quasipaa verrucospinosa

(Bourret, 1937)

2887F5FD-CC31-51A3-B550-751275B00C6A

##### Materials

- **Type status:** Other material. **Occurrence:** catalogNumber: VNUF R.2024. 39; individualCount: 1; sex: male; lifeStage: adult; occurrenceID: BADF3F23-3C2C-5B49-926F-504D0067E4DD; **Taxon:** genus: Quasipaa; specificEpithet: *verrucospinosa*; **Location:** country: Vietnam; stateProvince: Hanoi; municipality: Ba Vi; locality: Ba Vi National Park; verbatimElevation: 1000 m; verbatimCoordinates: 21 03.554N, 105 21.617E; verbatimCoordinateSystem: degrees decimal minutes; **Event:** eventDate: 29 July 2024; **Record Level:** institutionCode: VNUF
- **Type status:** Other material. **Occurrence:** catalogNumber: VNUF R.2024. 40; individualCount: 1; sex: female; lifeStage: adult; occurrenceID: 7DDCC09E-A5EC-5FAB-AFEB-BB3C92EC7875; **Taxon:** genus: Quasipaa; specificEpithet: *verrucospinosa*; **Location:** country: Vietnam; stateProvince: Hanoi; municipality: Ba Vi; locality: Ba Vi National Park; verbatimElevation: 1000 m; verbatimCoordinates: 21 03.554N, 105 21.617E; verbatimCoordinateSystem: degrees decimal minutes; **Event:** eventDate: 29 July 2024; **Record Level:** institutionCode: VNUF

##### Native status

VU (VNREDLIST 2024)

##### Conservation status

LC (IUCN 2025)

##### Distribution

In Vietnam, this species is known from Lao Cai (Hoang Lien National Park), Vinh Phuc (Tam Dao National Park), Ha Giang (Bac Me Nature Reserve) and Tuyen Quang (Na Hang Nature Reserve and Cham Chu Nature Reserve) Provinces, northern Vietnam (Pham et al. 2025). Elsewhere, this species has been reported from Laos (Frost 2026). First record in BVNP.

##### Notes

The adult male was found on the cliff and the female was found on the ground between 21:50 h and 22:10 h, both specimens found in the French Era Political Prison (ruins). The surrounding habitats were evergreen forest, streams and cliffs. Temperature was 27°C and humidity was 83%.

##### Diagnosis

Morphological characters of the specimens from BVNP agreed well with descriptions of Pham et al. (2025): SVL 78.8 mm in the male and 64.7 mm in the female); head wider than long (HW 35.5 mm, HL 33.2 mm in the male and HW 29.3 mm, HL 26.6 mm in the female); snout round; nostril oval, laterally positioned; tympanum distinct, supratympanic fold distinct; eyes large, pupils oval, iris green; vomerine teeth present; tongue notched posteriorly. Fore-limbs: short; finger free of webbing; male with nuptial pads spines present on finger I. Hind-limbs: long, robust; toes fully webbed. Skin: dorsal surface of head and body granular, back with large oval warts intermixed with small tubercles; sides of belly covered by oval tubercles with dark spines; ventral surface smooth. Colouration in life: dorsal surface yellowish-brown; dorsal limbs light brown with indistinct dark crossbars; throat and chest with black marbling; ventral surface of limbs, belly immaculate white (Fig. 3).

#### Megophryidae

D4A3063F-039B-53CE-A339-ABFB667B7A8C

#### Leptobrachella petrops

(Rowley, Dau, Hoang, Le, Cutajar & Nguyen, 2017)

CAC3FF2C-E9F5-5D7D-A3A4-3952EB8504EF

##### Native status

Not threatened

##### Conservation status

NT (IUCN 2025)

##### Distribution

This species is only known in Vietnam (Frost 2026). The record in BVNP by the report of Chen et al. (2018)

##### Notes

This species was observed during the present surveys.

#### Ophryophryne microstoma

Boulenger, 1903

7DDD91F0-B844-54F2-B6E6-4FAE4919F100

##### Materials

- **Type status:** Other material. **Occurrence:** catalogNumber: VNUF R.2024.25; individualCount: 1; sex: male; lifeStage: adult; occurrenceID: 087091B8-8EBC-5C75-AF45-F91240CAF6CE; **Taxon:** genus: Ophryophryne; specificEpithet: *microstoma*; **Location:** country: Vietnam; stateProvince: Hanoi; municipality: Ba Vi; locality: Ba Vi National Park; verbatimElevation: 382 m; verbatimCoordinates: 21 05.048N, 105 22.123E; verbatimCoordinateSystem: degrees decimal minutes; **Event:** eventDate: 11 July 2024; **Record Level:** institutionCode: VNUF
- **Type status:** Other material. **Occurrence:** catalogNumber: VNUF R.2024.31; individualCount: 1; sex: female; lifeStage: adult; occurrenceID: 8B56C207-5ECF-554D-A977-54BA47564F5B; **Taxon:** genus: Ophryophryne; specificEpithet: *microstoma*; **Location:** country: Vietnam; stateProvince: Hanoi; municipality: Ba Vi; locality: Ba Vi National Park; verbatimElevation: 828 m; verbatimCoordinates: 21 04.392N, 105 21.902E; verbatimCoordinateSystem: degrees decimal minutes; **Event:** eventDate: 13 July 2024; **Record Level:** institutionCode: VNUF

##### Native status

Not threatened

##### Conservation status

LC (IUCN 2025)

##### Distribution

In Vietnam, this species was known from Lao Cai and Ha Giang Provinces in the north, southwards to Dak Lak and Lam Dong Provinces. Elsewhere, this species has been reported from China, Laos, Thailand and Cambodia (Le et al. 2021). First record in BVNP.

##### Notes

The adult male VNUF R.2024.25 was found on the ground at Ngoc Hoa stream and the female VNUF R.2024.31 was found on the tree leaf at the ruins of Old French Church between 21:54 h and 23:00 h. The surrounding habitats were secondary forest, streams and banana forest. The relative temperature was 24–27°C and humidity was 92–94%.

##### Diagnosis

Morphological characters of the specimens from BVNP agreed well with descriptions of Pham et al. (2020) and Le et al. (2021): Size small (SVL 39.0 mm in the male and 38.0 mm in the female); head small, wider than long (HW 11.6 mm, HL 11.4 mm in the male and HW 11.2 mm, HL 11.0 mm in the female); snout truncate, protruding, projecting beyond upper jaw; tympanum round, distinct, supratympanic fold distinct; vomerine teeth absent; tongue round; pupil diamond-shaped, upper eyelid with a dermal horn on outer edge. Fore-limbs: slender; finger free of webbing; male with nuptial pads spines present on fingers I and II. Hind-limbs: slender; toe webbing rudimental. Skin: dorsal with small warts, with symmetric glandular ridges; flanks covered with granules; ventral surface smooth. Colouration in life: dorsum reddish-brown, upper surface of limbs with transverse bars; a light bar present below the eye; flanks with some small black spots; ventral surface cream with dark marbling (Fig. 3).

#### Xenophrys maosonensis

(Bourret, 1937)

1D9C2FA1-55CF-5273-B441-FFBAFD6D8181

##### Native status

Not threatened

##### Conservation status

DD (IUCN 2025)

##### Distribution

Vietnam (Quang Thanh, Cao Bang, Ba Vi: Nguyen et al. (2009), Tam Dao, Vinh Phuc, Sa Pa, Lao Cai, Quang Ninh and Son La Provinces) and China (Frost 2026).

##### Notes

Previously recorded from BVNP by Nguyen et al. (2009), observed during the present surveys.

#### Microhylidae

BEF75FF4-3B1F-5A22-8DEC-1B946F92E524

#### Kaloula pulchra

Gray, 1831

1553926F-E941-5098-BDA6-38D7D25C135C

##### Materials

- **Type status:** Other material. **Occurrence:** catalogNumber: IB A.2025.050; individualCount: 1; sex: male; lifeStage: juvenile; occurrenceID: DCE94630-6CCA-51DF-BE0A-A221905B3594; **Taxon:** genus: Kaloula; specificEpithet: *pulchra*; **Location:** country: Vietnam; stateProvince: Hanoi; municipality: Ba Vi; locality: Ba Vi National Park; verbatimElevation: 250 m; verbatimCoordinates: 21 05.177N, 105 23.201E; verbatimCoordinateSystem: degrees decimal minutes; **Event:** eventDate: 21 June 2025; **Record Level:** institutionCode: IB
- **Type status:** Other material. **Occurrence:** catalogNumber: IB A.2025.055; individualCount: 1; sex: female; lifeStage: juvenile; occurrenceID: 4FEC4E80-A407-5224-8D9C-C589C09B3A31; **Taxon:** genus: Kaloula; specificEpithet: *pulchra*; **Location:** country: Vietnam; stateProvince: Hanoi; municipality: Ba Vi; locality: Ba Vi National Park; verbatimElevation: 250 m; verbatimCoordinates: 21 05.177N, 105 23.201E; verbatimCoordinateSystem: degrees decimal minutes; **Event:** eventDate: 21 June 2025; **Record Level:** institutionCode: IB

##### Native status

Not threatened

##### Conservation status

LC (IUCN 2025)

##### Distribution

In Vietnam, this species has been recorded from Bac Kan and Thai Nguyen Provinces in the north, southwards to Tay Ninh and Ca Mau Provinces. Elsewhere, this species has been recorded from Bangladesh, Cambodia, China, India, Indonesia, Laos, Myanmar, the Philippines, Singapore,Taiwan and Thailand (Luong et al. 2025).

##### Notes

The specimens were found at 21:00 h on the main road. The surrounding habitat was evergreen forest. The relative temperature was 26°C and humidity was 95%.

##### Diagnosis

Morphological characters of the specimen from BVNP agreed well with descriptions of Luong et al. (2025): Size small (SVL 32.0 mm in both sexes); head shorter than wide (HL 9.4 mm, HW 11.1 mm in male and HL 9.0 mm, HW 11.3 mm in female); snout round; nostrils round; canthus rostralis scarcely indicated, loreal region sloping obliquely, not concave; tympanum indistinct; vomerine teeth present; tongue notched posteriorly. Fore-limbs: Arms short; tips of fingers enlarged; fingers free of webbing. Hind-limbs: Thigh short; tips of toes not enlarged; webbing rudimentary. Skin: dorsal surface of head and body finely granular with some scattered pustular tubercles, ventral surface granulate. Colouration in life: dorsal surface of head and body dark brown; an irregularly-edged light brown diagonal stripe from eye to groin; flanks dark brown; Dorsal surface of limbs with some light brown pattern; ventral surface light grey with grey markings (Fig. 3).

#### Microhyla butleri

Boulenger, 1900

B1B9C062-7F5C-5126-AC78-0A5849E47035

##### Materials

- **Type status:** Other material. **Occurrence:** catalogNumber: IB A.2025.044; individualCount: 1; sex: male; lifeStage: adult; occurrenceID: DF2BF7AA-4913-583B-973D-8FDDCC91E504; **Taxon:** genus: Microhyla; specificEpithet: *butleri*; **Location:** country: Vietnam; stateProvince: Hanoi; municipality: Ba Vi; locality: Ba Vi National Park; verbatimElevation: 250 m; verbatimCoordinates: 21 05.177N, 105 23.201E; verbatimCoordinateSystem: degrees decimal minutes; **Event:** eventDate: 21 June 2025; **Record Level:** institutionCode: IB
- **Type status:** Other material. **Occurrence:** catalogNumber: IB A.2025.043; individualCount: 1; sex: female; lifeStage: adult; occurrenceID: 3D1CFF4A-4CD7-5905-87B1-2D3C3812B56F; **Taxon:** genus: Microhyla; specificEpithet: *butleri*; **Location:** country: Vietnam; stateProvince: Hanoi; municipality: Ba Vi; locality: Ba Vi National Park; verbatimElevation: 250 m; verbatimCoordinates: 21 05.177N, 105 23.201E; verbatimCoordinateSystem: degrees decimal minutes; **Event:** eventDate: 21 June 2025; **Record Level:** institutionCode: IB

##### Native status

Not threatened

##### Conservation status

LC (IUCN 2025)

##### Distribution

This is a widespread species in Vietnam, except for the Mekong Delta. Elsewhere, this species has been reported from China, Myanmar, Thailand, Malaysia and Singapore (Nguyen et al. 2009). First record in BVNP.

##### Notes

The specimens were found at 21:00 h on the main road. The surrounding habitat was evergreen forest. The relative temperature was 26°C and humidity was 95%.

##### Diagnosis

Morphological characters of the specimen from BVNP agreed well with descriptions of Pham et al. (2020): Size small (SVL 20.0 mm in male and 25.5 mm in female); head shorter than wide (HL 5.4 mm, HW 6.5 mm in male and HL 7.0 mm, HW 7.5 mm in female); snout round, pronounced; tympanum invisible; vomerine teeth absent; tongue roundly spatulate. Fore-limb slender; fingers free of webbing, with slightly developed discs. Hind-limb slender; toes with small discs, basally webbed. Skin: dorsal skin with tiny tubercles on the dorsum and tibia; ventral skin smooth, cloacal region granular. Colouration in life: dorsal surface of head grey, dorsal surface of body brown; a whitish stripe extending from eye to shoulder; limbs with dark transverse bars; ventral surface whitish (Fig. 3).

#### Microhyla cf. heymonsi

Vogt, 1911

8D581A18-47EF-5863-ABE2-892485C95AB2

##### Materials

- **Type status:** Other material. **Occurrence:** catalogNumber: IB A.2025.021; individualCount: 1; sex: male; lifeStage: adult; occurrenceID: 1419F3B3-20DD-5B6E-B88A-B787F46339FB; **Taxon:** genus: Microhyla; specificEpithet: cf. *heymonsi*; **Location:** country: Vietnam; stateProvince: Hanoi; municipality: Ba Vi; locality: Ba Vi National Park; verbatimElevation: 250 m; verbatimCoordinates: 21 05.177N, 105 23.201E; verbatimCoordinateSystem: degrees decimal minutes; **Event:** eventDate: 15 June 2025; **Record Level:** institutionCode: IB
- **Type status:** Other material. **Occurrence:** catalogNumber: IB A.2025.026; individualCount: 1; sex: female; lifeStage: adult; occurrenceID: 75E3DE28-38F8-5FB8-BB63-C0D2D11988CC; **Taxon:** genus: Microhyla; specificEpithet: cf. *heymonsi*; **Location:** country: Vietnam; stateProvince: Hanoi; municipality: Ba Vi; locality: Ba Vi National Park; verbatimElevation: 250 m; verbatimCoordinates: 21 05.177N, 105 23.201E; verbatimCoordinateSystem: degrees decimal minutes; **Event:** eventDate: 15 June 2025; **Record Level:** institutionCode: IB

##### Native status

Not threatened

##### Conservation status

LC (IUCN 2025)

##### Distribution

In Vietnam, this species is known from Lao Cai and Ha Giang Provinces in the north, southwards to Kien Giang and Ca Mau Provinces. Elsewhere, this species has been reported from from China, Taiwan, Cambodia, Laos, Myanmar, Thailand and Malaysia (Pham et al. 2025). First record in BVNP.

##### Notes

The specimens were found at 21:00 h on the main road. The surrounding habitat was evergreen forest. The relative temperature was 26°C and humidity was 95%.

##### Diagnosis

Morphological characters of the specimen from BVNP agreed well with descriptions of Pham et al. (2025): Size small (SVL 25.4 mm in male and 27.0 mm in female); head longer than wide (HL 7.0 mm, HW 6.0 mm in male and HL 8.0 mm, HW 7.4 mm in female); tympanum invisible; vomerine teeth absent; tongue roundly spatulate. Fore-limb slender; fingers free of webbing and tips not swollen. Hind-limb slender; toe tips round, not swollen, webbed at base. Skin: dorsal skin smooth, dorsolateral edges not sharp; ventral skin smooth. Colouration in life: dorsal surface of head and body pale grey, with a white stripe from tip of snout to cloaca and a small dark spot in the centre of the back; lateral side of head and flank dark brown to black; anterior part of thighs, cloacal region and lower parts of feet black; limbs with thin transverse bars; ventral surface white to grey (Fig. 3).

#### Microhyla mukhlesuri

Hasan, Islam, Kuramoto, Kurabayashi & Sumida, 2014

C51838F7-319B-56D0-9586-FD850BB55AA0

##### Materials

- **Type status:** Other material. **Occurrence:** catalogNumber: IB A.2024.104; individualCount: 1; sex: male; lifeStage: adult; occurrenceID: 0AA9458B-4B11-537C-817B-B691D8C19E59; **Taxon:** genus: Microhyla; specificEpithet: *mukhlesuri*; **Location:** country: Vietnam; stateProvince: Phu Tho; municipality: Ky Son; locality: Ba Vi National Park; verbatimElevation: 150 m; verbatimCoordinates: 20 55.913N, 105 26.608E; verbatimCoordinateSystem: degrees decimal minutes; **Record Level:** institutionCode: IB

##### Native status

Not threatened

##### Conservation status

LC (IUCN 2025)

##### Distribution

In Vietnam, this species has been recorded from Lao Cai and Dien Bien Provinces in the north, southwards to Quang Nam Province. Elsewhere, this species has been reported from China, Myanmar, Laos, Cambodia, Thailand, India, Bangladesh and Malaysia (Pham et al. 2025). First record in BVNP.

##### Notes

The specimen was found at 23:00 h on the ground. The surrounding habitats were streams and shrubs. The relative temperature was 25°C and humidity was 93%.

##### Diagnosis

Morphological characters of the specimen from BVNP agreed well with descriptions of Pham et al. (2025): Size small (SVL 20.5 mm); head longer than wide (HL 7.4 mm, HW 6.0 mm); snout round; tympanum invisible; vomerine teeth absent; tongue roundly spatulate; Fore-limb slender; fingers free of webbing and tips not swollen. Hind-limb slender; toe tips round, not swollen, webbed at base. Skin: dorsal skin smooth, dorsolateral ridges discontinuous; ventral skin smooth. Colouration in life: dorsal surface of head and body grey with a dark X-shaped mark on the dorsum, arising from the eyes to the groin; limbs with dark transverse bars; ventral surface whitish, throat and chest mottled with dark brown (Fig. 3).

#### Microhyla pulchra

(Hallowell, 1861)

B47448DB-CE40-55DB-BF13-C609344C676C

##### Materials

- **Type status:** Other material. **Occurrence:** catalogNumber: VNUF R.2024.46; individualCount: 1; sex: female; lifeStage: adult; occurrenceID: 2E9D41DF-3438-5E2C-9FF2-5C5EBA045091; **Taxon:** genus: Microhyla; specificEpithet: *pulchra*; **Location:** country: Vietnam; stateProvince: Hanoi; municipality: Ba Vi; locality: Ba Vi National Park; verbatimElevation: 150 m; verbatimLatitude: 21°05.060’N; verbatimLongitude: 105°22.576’E; verbatimCoordinateSystem: degrees decimal minutes; **Event:** eventDate: 15 July 2024; **Record Level:** institutionCode: VNUF

##### Native status

Not threatened

##### Conservation status

LC (IUCN 2025)

##### Distribution

This is a common species in Vietnam. Elsewhere, this species has been reported from India, China, Cambodia, Laos and Thailand (Nguyen et al. 2009). First record in BVNP.

##### Notes

The specimen was found on the main road in early morning 00:10 h. The surrounding habitat was the pine forest. The relative temperature was 26°C and humidity was 91%.

##### Diagnosis

Morphological characters of the specimens from BVNP agreed well with the descriptions of Hecht et al. (2013) and Pham et al. (2020): Size small (SVL 33.1 mm); body flattened, triangle-shaped; head slightly wider than long (HW 9.7 mm, HL 8.3 mm); snout obtusely pointed, slightly pronounced; tympanum indistinct; vomerine teeth absent; tongue round posteriorly. Fore-limbs: short and slender; fingers free of webbing. Hind-limbs: fatter. Skin: dorsum and venter smooth; a distinct fold present between posterior edges of the eyes; cloacal region granular. Colouration in life: dorsum brown with a dark band between eyes; several V-shaped dark and lighter alternating bands on dorsum pointing to the head; dark transversal band from posterior corner of the eye towards upper flanks; rear of flanks and inner parts of legs yellow; limbs with transverse bars; ventral surface whitish yellow; throat and chest speckled with grey (Fig. 3).

#### Nanohyla marmorata

(Bain and Nguyen, 2004)

70A45FF0-430F-546B-992A-AA50C910A273

##### Materials

- **Type status:** Other material. **Occurrence:** catalogNumber: VNUF R.2024.30; individualCount: 1; sex: male; lifeStage: adult; occurrenceID: 7BD3918C-7CEE-52B7-B150-3643A23A7BB8; **Taxon:** genus: Nanohyla; specificEpithet: *marmorata*; **Location:** country: Vietnam; stateProvince: Hanoi; municipality: Ba Vi; locality: Ba Vi National Park; verbatimElevation: 828 m; verbatimCoordinates: 21 04.392N, 105 21.902E; verbatimCoordinateSystem: degrees decimal minutes; **Event:** eventDate: 13 July 2024; **Record Level:** institutionCode: VNUF
- **Type status:** Other material. **Occurrence:** catalogNumber: VNUF R.2024.85; individualCount: 1; sex: female; lifeStage: adult; occurrenceID: CD041AA8-27E8-5F78-965E-3E482885551B; **Taxon:** genus: Nanohyla; specificEpithet: *marmorata*; **Location:** country: Vietnam; stateProvince: Hanoi; municipality: Ba Vi; locality: Ba Vi National Park; verbatimElevation: 1000 m; verbatimCoordinates: 21 03.588N, 105 21.693E; verbatimCoordinateSystem: degrees decimal minutes; **Event:** eventDate: 21 July 2024; **Record Level:** institutionCode: VNUF

##### Native status

Not threatened

##### Conservation status

LC (IUCN 2025)

##### Distribution

In Vietnam, this species is known from Ha Tinh, Quang Binh, Quang Tri, Quang Nam, Kon Tum, Thanh Hoa, Gia Lai, Binh Dinh and Thua Thien-Hue Provinces. Elsewhere, this species has been reported from Laos (Frost 2026). First record in BVNP.

##### Notes

The adult male VNUF R.2024.30 was found on the tree leaf at 21:54 h in the ruins of Old French Church with the surrounding habitat being secondary forest, temperature 24°C and humidity 92%. The female VNUF R.2024.85 was found on the ground at 23:27 h in the French Era Political Prison (ruins) with the surrounding habitats being evergreen forest and streams, temperature 25°C and humidity 88%.

##### Diagnosis

Morphological characters of the specimens from BVNP agreed well with descriptions of Bain and Nguyen (2004): Body slender, flattened, triangular-shaped (SVL 19.5 mm in the male and 22.4 mm in the female); head wider than long (HW 6.4 mm, HL 6.8 mm in the male and HW 7.0 mm, HL 7.2 mm in the female); snout round; nostrils oval; tympanum indistinct; vomerine teeth absent; tongue roundly spatulate. Fore-limbs: slender; fingers free of webbing. Hind-limbs: slender. Skin: dorsolateral skin, flanks and, on the upper part of the limbs, small pimples; ventral smooth. Colouration in life: dorsum grey brown; dorsal head with a butterfly pattern between eyes; white stripe running from lower posterior corner of eye to jaw; limbs banded, legs with one band distinctly prominent; chin black; throat, chest mottled brown; belly with distinctive large white-brown marbling (Fig. 3).

#### Vietnamophryne cuongi

Nguyen, Hoang, Jiang, Orlov, Ninh, Nguyen, Nguyen & Ziegler, 2021

2F9EF3FF-E43E-5C73-8615-E59F26BC662D

##### Native status

Not threatened

##### Conservation status

Not evaluated

##### Distribution

In Vietnam, this species only known from BVNP (Nguyen et al. 2021, Frost 2026).

##### Notes

Previously recorded from BVNP by Nguyen et al. (2021), observed during the present surveys.

#### Ranidae

FF3C30B5-D346-5550-AEBC-2656394E663F

#### Hylarana guentheri

(Boulenger, 1882)

107EA800-2734-5CDE-9E30-A66DA9F4611B

##### Native status

Not threatened

##### Conservation status

LC (IUCN 2025)

##### Distribution

Vietnam (entire country: Nguyen et al. (2009)), China, Taiwan, Myanmar and Laos (Nguyen et al. 2009).

##### Notes

Previously recorded from BVNP by Nguyen et al. (2009), observed during the present surveys.

#### Hylarana maosonensis

Bourret, 1937

D4AA5BB9-5F28-5837-A3D7-43110F939E90

##### Materials

- **Type status:** Other material. **Occurrence:** catalogNumber: VNUF R.2024. 60; individualCount: 1; sex: male; lifeStage: adult; occurrenceID: CB8B8A52-B382-5662-A8B4-8365BD08C940; **Taxon:** genus: Hylarana; specificEpithet: *maosonensis*; **Location:** country: Vietnam; stateProvince: Hanoi; municipality: Ba Vi; locality: Ba Vi National Park; verbatimElevation: 1051 m; verbatimCoordinates: 21 03.589N, 105 21.693E; verbatimLatitude: 21°03.589’N; verbatimLongitude: 105°21.693’E; verbatimCoordinateSystem: degrees decimal minutes; **Event:** eventDate: 19 July 2024; **Record Level:** institutionCode: VNUF
- **Type status:** Other material. **Occurrence:** catalogNumber: VNUF R.2024.61; individualCount: 1; sex: male; lifeStage: adult; occurrenceID: F591BF94-FEC8-57F9-BB66-0BE4FAE9E47A; **Taxon:** genus: Hylarana; specificEpithet: *maosonensis*; **Location:** country: Vietnam; stateProvince: Hanoi; municipality: Ba Vi; locality: Ba Vi National Park; verbatimElevation: 1051 m; verbatimCoordinates: 21 03.589N, 105 21.693E; verbatimLatitude: 21°03.589’N; verbatimLongitude: 105°21.693’E; verbatimCoordinateSystem: degrees decimal minutes; **Event:** eventDate: 19 July 2024; **Record Level:** institutionCode: VNUF
- **Type status:** Other material. **Occurrence:** catalogNumber: VNUF R.2024.59; individualCount: 1; sex: female; lifeStage: adult; occurrenceID: F15723B9-AA3A-56A3-BBA3-861824B6012A; **Taxon:** genus: Hylarana; specificEpithet: *maosonensis*; **Location:** country: Vietnam; stateProvince: Hanoi; municipality: Ba Vi; locality: Ba Vi National Park; verbatimElevation: 1051 m; verbatimCoordinates: 21 03.589N, 105 21.693E; verbatimLatitude: 21°03.589’N; verbatimLongitude: 105°21.693’E; verbatimCoordinateSystem: degrees decimal minutes; **Event:** eventDate: 19 July 2024; **Record Level:** institutionCode: VNUF

##### Native status

Not threatened

##### Conservation status

LC (IUCN 2025)

##### Distribution

In Vietnam, this species is known from Tuyen Quang, Cao Bang, Yen Bai, Vinh Phu, Bac Thai, Bac Khan, Ha Bac, Quang Ninh, Bac Giang, Hai Hung, Ha Tay, Tanh Hoa, Son La, Ninh Binh and Hoa Binh Provinces) and central (Quang Binh Province). Elsewhere, this species has been reported from China and Laos (Frost 2026). First record in BVNP.

##### Notes

Specimens were found between 22:30 h and 23:40 h on the tree leaves in the French Era Political Prison (ruins). The surrounding habitats were evergreen forest and streams. The relative temperature was 23–25°C and humidity was 85–91%.

##### Diagnosis

Morphological characters of the specimens from BVNP agreed well with the description of Hecht et al. (2013) and Pham et al. (2020): Size medium (SVL 39.0–43.5 mm in males and 53.2 mm in the female); head longer than wide (HL 14.3–19.6 mm, HW 12.4–14.4 mm in males and HL 21.4 mm in the female, HW 20.1 mm in the female); snout round; tympanum distinct; vomerine teeth present; tongue notched posteriorly. Fore-limbs: fingers free of webbing; males with nuptial pad on finger I. Hind-limbs: toes with webbing. Skin: dorsal surface of head, body, flanks and thighs with tubercles; dorsolateral fold distinct; supratympanic fold prominent; venter smooth. Colouration in life: dorsum yellowish-brown, flanks lighter with black spots; limbs with dark brown transverse bars; tympanum brown; venter whitish (Fig. 3).

#### Hylarana taipehensis

(Van Denburgh, 1909)

F6F78A80-2E65-51AB-ACE8-5E2EF74DD793

##### Native status

Not threatened

##### Conservation status

LC (IUCN 2025)

##### Distribution

Vietnam (Ba Vi: Nguyen et al. (2009)), India, Nepal, Bangladesh, China, Myanmar, Laos, Thailand and Cambodia (Nguyen et al. 2009).

##### Notes

Previously recorded from BVNP by Nguyen et al. (2009), not observed during the present surveys.

#### Odorrana chloronota

(Günther, 1876)

774D3196-FA59-5782-9DD1-805C1D7C80B6

##### Materials

- **Type status:** Other material. **Occurrence:** catalogNumber: IB A.2025.071; individualCount: 1; sex: male; lifeStage: juvenile; occurrenceID: 49DC1B3B-79E9-5125-BB99-0E89EE2CBAE4; **Taxon:** genus: Odorrana; specificEpithet: *chloronota*; **Location:** country: Vietnam; stateProvince: Hanoi; municipality: Ba Vi; locality: Ba Vi National Park; verbatimElevation: 900 m; verbatimCoordinates: 21 04.331N, 105 21.752E; verbatimCoordinateSystem: degrees decimal minutes; **Event:** eventDate: 29 June 2025; **Record Level:** institutionCode: IB
- **Type status:** Other material. **Occurrence:** catalogNumber: IB A.2025.070; individualCount: 1; sex: female; lifeStage: juvenile; occurrenceID: 791DB94C-48BC-55C1-8738-872ABA28544E; **Taxon:** genus: Odorrana; specificEpithet: *chloronota*; **Location:** country: Vietnam; stateProvince: Hanoi; municipality: Ba Vi; locality: Ba Vi National Park; verbatimElevation: 900 m; verbatimCoordinates: 21 04.331N, 105 21.752E; verbatimCoordinateSystem: degrees decimal minutes; **Event:** eventDate: 29 June 2025; **Record Level:** institutionCode: IB

##### Native status

Not threatened

##### Conservation status

LC (IUCN 2025)

##### Distribution

In Vietnam, this species has been reported from Bac Kan, Lang Son, Vinh Phuc, Quang Ninh and Lam Dong Provinces. Elsewhere, the species has been reported from north-eastern India and Myanmar to southern China (Luong et al. 2021). First record in BVNP.

##### Notes

The specimens were found on the main road at 22:00 h. The surrounding habitat was evergreen forest. The relative temperature was 25°C and the humidity was 90%.

##### Diagnosis

Morphological characters of the specimens from BVNP agreed well with descriptions of Luong et al. (2021): Male smaller than female (SVL 38.5 mm in male and 42.7 mm in female); head longer than wide (HL 15.3 mm, HW 13.0 mm in male and HL 16.0 mm, HW 14.0 mm in femlae); snout round; pupil round; tympanum distinct; vomerine teeth present; tongue cordiform. Fore-limbs: slender, tips of fingers enlarged into discs; fingers free of webbing. Hind-limbs: slender, tips of toes enlarged into discs; webbing complete. Skin: dorsal surface of head and body smooth; flank with small tubercles, dorsolateral fold absent; ventral surface smooth. Colouration in life: dorsum green with black spots; lateral side of head and flanks brownish-grey; lips white; hind-limbs with distinct dark brown bars; webbing dark grey; venter white (Fig. 3).

#### Rhacophoridae

FD45DE29-1B53-5600-B652-DA0F13A3A038

#### Kurixalus bisacculus

(Taylor, 1962)

6A7D943D-CA1C-5CF4-BF2B-34275BCE91CB

##### Materials

- **Type status:** Other material. **Occurrence:** catalogNumber: IB A.2025.001; individualCount: 1; sex: male; lifeStage: adult; occurrenceID: 4DFE72D1-EEA2-56FA-967D-8FB649C5D99D; **Taxon:** genus: Kurixalus; specificEpithet: *bisacculus*; **Location:** country: Vietnam; stateProvince: Hanoi; municipality: Ba Vi; locality: Ba Vi National Park; verbatimElevation: 1000 m; verbatimCoordinates: 21 03.658N, 105 21.737E; verbatimCoordinateSystem: degrees decimal minutes; **Event:** eventDate: 11 June 2025; **Record Level:** institutionCode: IB
- **Type status:** Other material. **Occurrence:** catalogNumber: IB A.2025.002; individualCount: 1; sex: male; lifeStage: adult; occurrenceID: 4DFE72D1-EEA2-56FA-967D-8FB649C5D99D; **Taxon:** genus: Kurixalus; specificEpithet: *bisacculus*; **Location:** country: Vietnam; stateProvince: Hanoi; municipality: Ba Vi; locality: Ba Vi National Park; verbatimElevation: 1000 m; verbatimCoordinates: 21 03.658N, 105 21.737E; verbatimCoordinateSystem: degrees decimal minutes; **Event:** eventDate: 11 June 2025; **Record Level:** institutionCode: IB

##### Native status

Not threatened

##### Conservation status

LC (IUCN 2025)

##### Distribution

In Vietnam, this species is known from Lao Cai, Cao Bang and Ha Giang in the north, southwards to Thua Thien-Hue and Gia Lai Provinces (Hecht et al. 2013). Elsewhere, this species has been reported from from Cambodia, China, Laos, Myanmar and Thailand (Frost 2026). First record in BVNP.

##### Notes

Both specimens were found at 23:40 h on a tree leaf along the Thuong Temple trail. The surrounding habitat was evergreen forest. The relative temperature was 21°C and humidity was 90%.

##### Diagnosis

Morphological characters of the specimens from BVNP agreed well with descriptions of Pham et al. (2017): SVL 30.0–31.0 mm; head longer than wide (HL 11.3–11.5 mm, HW 13.0 mm in male and HL 16.0 mm, HW 14.0 mm in female); snout pointed anteriorly; pupil round; tympanum distinct; vomerine teeth present; tongue notched. Fore-limbs: slender, tips of fingers enlarged into discs; fingers free of webbing. Hind-limbs: slender, tips of toes enlarged into discs; webbing complete. Skin: some scattered flat tubercles on the head, eyelids and occiput, sparse on dorsum, more dense and larger on flanks, fine granules on rump; chin granular, chest nearly smooth; venter and lower part of sides granular. Colouration in life: dorsal head and body light brown with green marking, occiput with a dark green marking in triangular shape; tympanum brownish; chin cream with dark spots; throat, chest, venter and underside of limbs cream (Fig. 3).

#### Polypedates megacephalus

Hallowell, 1861

2189BD7F-DCC0-5026-BD56-485E4F631452

##### Native status

Not threatened

##### Conservation status

LC (IUCN 2025)

##### Distribution

Vietnam (Ba Vi: Nguyen et al. (2009)), India, China, Myanmar, Taiwan, Laos, Thailand and Japan (Nguyen et al. 2009).

##### Notes

Previously recorded from BVNP by Nguyen et al. (2009), observed during the present surveys.

#### Polypedates mutus

(Smith, 1940)

4C6D8D5B-0149-53BF-B671-15FC3BA0D980

##### Native status

Not threatened

##### Conservation status

LC (IUCN 2025)

##### Distribution

Vietnam (Ba Vi: Nguyen et al. (2009)), China, Myanmar, Laos, and Thailand (Nguyen et al. 2009).

##### Notes

Previously recorded from BVNP by Nguyen et al. (2009), observed during the present surveys.

#### Raorchestes parvulus

(Boulenger, 1893)

D61A9736-C47E-5A68-8FFF-47BB0662D660

##### Materials

- **Type status:** Other material. **Occurrence:** catalogNumber: VNUF R.2024.48; individualCount: 1; sex: male; lifeStage: adult; occurrenceID: 867F40CC-D27D-5385-BD0F-BC52C9BB15C3; **Taxon:** genus: Raorchestes; specificEpithet: *parvulus*; **Location:** country: Vietnam; stateProvince: Hanoi; municipality: Ba Vi; locality: Ba Vi National Park; verbatimElevation: 1025 m; verbatimCoordinates: 21 03.613N, 105 21.728E; verbatimCoordinateSystem: degrees decimal minutes; **Event:** eventDate: 18 April 2024; **Record Level:** institutionCode: VNUF
- **Type status:** Other material. **Occurrence:** catalogNumber: VNUF R.2024.49; individualCount: 1; sex: male; lifeStage: adult; occurrenceID: B158299D-F44F-59A8-B975-4E7CB96FEA00; **Taxon:** genus: Raorchestes; specificEpithet: *parvulus*; **Location:** country: Vietnam; stateProvince: Hanoi; municipality: Ba Vi; locality: Ba Vi National Park; verbatimElevation: 1025 m; verbatimCoordinates: 21 03.613N, 105 21.728E; verbatimCoordinateSystem: degrees decimal minutes; **Event:** eventDate: 18 April 2024; **Record Level:** institutionCode: VNUF

##### Native status

Not threatened

##### Conservation status

LC (IUCN 2025)

##### Distribution

In Vietnam, this species has been recorded from Vinh Phu, Bac Thai Lao Cai, Lai Chau, Son La and Tuyen Quang Provinces. Elsewhere, this species has been reported from Bangladesh, Cambodia, Laos, Malaysia, Myanmar and Thailand (Frost 2026). First record in BVNP.

##### Notes

Both specimens were found between 22:30 h and 23:40 h on tree leaves. The surrounding habitats were evergreen forest and streams. The relative temperature was 23–25°C and humidity was 85–91%.

##### Diagnosis

Morphological characters of the specimens from BVNP agreed well with descriptions of Le et al. (2021)andPham et al. (2022): Body size small (SVL 22.2‒23.3 mm); head slightly longer than wide (HL 7.5–8.5 mm, HW 7.2–7.6 mm); snout rounded; vomerine teeth present; tongue notched posteriorly; both of specimens have large external vocal sac. Fore-limbs: Forearm short; finger free of webbing. Hind-limbs: short; toes with webbing. Skin: dorsal surface with small, scattered, conical warts; ventral surface granular. Colouration in life: dorsal greyish, with a triangular blotch between the eyes and a butterfly-like dark band on the back; belly and lower surface of the upper arm granular, whitish (Fig. 3).

#### Theloderma corticale

(Boulenger, 1903)

4CED3512-A2FE-5E32-80B8-4513D158C360

##### Materials

- **Type status:** Other material. **Occurrence:** catalogNumber: VNUF R.2024.01; individualCount: 1; sex: male; lifeStage: adult; occurrenceID: 2AAD69F1-27BE-5C15-86B9-0C9E074BB9E3; **Taxon:** genus: Theloderma; specificEpithet: *corticale*; **Location:** country: Vietnam; stateProvince: Hanoi; municipality: Ba Vi; locality: Ba Vi National Park; verbatimElevation: 400 m; verbatimCoordinates: 21 05.069N, 105 22.567E; verbatimCoordinateSystem: degrees decimal minutes; **Event:** eventDate: 15 March 2024; **Record Level:** institutionCode: VNUF
- **Type status:** Other material. **Occurrence:** catalogNumber: VNUF R.2024.02; individualCount: 1; sex: male; lifeStage: adult; occurrenceID: 89E157F0-1B9D-578F-9EB1-F4335776DE06; **Taxon:** genus: Theloderma; specificEpithet: *corticale*; **Location:** country: Vietnam; stateProvince: Hanoi; municipality: Ba Vi; locality: Ba Vi National Park; verbatimElevation: 400 m; verbatimCoordinates: 21 05.069N, 105 22.567E; verbatimCoordinateSystem: degrees decimal minutes; **Event:** eventDate: 15 March 2024; **Record Level:** institutionCode: VNUF

##### Native status

VU (VNREDLIST 2024)

##### Conservation status

LC (IUCN 2025)

##### Distribution

In Vietnam, this species is known from Bac Giang, Bac Kan, Bac Thai, Cao Bang, Ha Giang [Du Gia], Hoa Binh, Kon Tum, Lang Son [Mau Son], Ninh Binh (Cuc Phuong), Quang Binh (Da Lat Forest, Thuong Hoa Commune, Minh Hoa District, Cha Noi forest, Xuan Trach Commune and Bo Trach District), Quang Tri, Son La [Muong Do], Thai Nguyen, Tuyen Quang (Na Hang, Son Duong), Vinh Phuc (Tam Dao), Thanh Hoa and Bac Giang Provinces. Elsewhere, this species has been reported from China and Laos (Frost 2026). First record in BVNP.

##### Notes

Both of specimens were found near the water tank of the ranger station between 22:00 h and 23:00 h. The relative temperature was 23°C and humidity was 97%.

##### Diagnosis

Morphological characters of the specimens from BVNP agreed well with descriptions of Luu et al. (2013) and Pham et al. (2020): SVL 58.4–69.0 mm; head wider than long (HW 26.5–29.4 mm, HL 25.2–27.1 mm); snout round; tympanum oval; vomerine teeth present; tongue notched posteriorly. Fore-limbs: fingers free of webbing; tips of fingers enlarged into round discs; nuptial pad present on finger I. Hind-limbs, tips of toes enlarged into round discs; toes with webbing. Skin: dorsal surface of head, body and limbs covered with tubercles or warts of different sizes; ventral skin with small tubercles. Colouration in life: dorsal surface green marbled with reddish-brown spots; dark brown bars present on upper surface of fore- and hind-limbs; ventral surface yellow with green marbling (Fig. 3).

#### Ichthyophiidae

5CBE04E4-4524-5F6A-A048-148EE8FCEFC4

#### Ichthyophis kohtaoensis

Taylor, 1960

83866D6B-4A61-5E01-B968-034C57E54BFF

##### Materials

- **Type status:** Other material. **Occurrence:** catalogNumber: IB A.2025.024; individualCount: 1; sex: female; lifeStage: adult; occurrenceID: 57E08A9D-2E51-518C-A7D7-903E986982DB; **Taxon:** genus: Ichthyophis; specificEpithet: *kohtaoensis*; **Location:** country: Vietnam; stateProvince: Hanoi; municipality: Ba Vi; locality: Ba Vi National Park; verbatimElevation: 500 m; verbatimCoordinates: 21 04.952N, 105 22.283E; verbatimCoordinateSystem: degrees decimal minutes; **Event:** eventDate: 15 June 2025; **Record Level:** institutionCode: IB
- **Type status:** Other material. **Occurrence:** catalogNumber: IB A.2025.058; individualCount: 1; sex: male; lifeStage: juvenile; occurrenceID: F8FBBB9A-5B58-5693-8896-077E6CB9113E; **Taxon:** genus: Ichthyophis; specificEpithet: *kohtaoensis*; **Location:** country: Vietnam; stateProvince: Hanoi; municipality: Ba Vi; locality: Ba Vi National Park; verbatimElevation: 250 m; verbatimCoordinates: 21 05.177N, 105 23.201E; verbatimCoordinateSystem: degrees decimal minutes; **Event:** eventDate: 21 June 2025; **Record Level:** institutionCode: IB

##### Native status

Not threatened

##### Conservation status

DD (IUCN 2025)

##### Distribution

In Vietnam, this species is known from Cao Bang and Tuyen Quang Provinces south to Hoa Binh, Dong Thap Provinces and possibly to Ca Cau Province. Elsewhere, this species has been reported from Cambodia, China, Laos, Myanmar and Thailand (Frost 2026). First record in BVNP.

##### Notes

Both of specimens were found on the main road at 22:00 h. The surrounding habitat was evergreen forest. The relative temperature was 23°C and humidity was 95%.

##### Diagnosis

Morphological characters of the specimens from BVNP agreed well with the descriptions of Hecht et al. (2013) and Ngo et al. (2022): TL 305.0 mm in female and 250.0 mm in juvenile male; mid-body width (BWM 15.3 mm in female and 13.15 mm in juvenile male); head length (HL 15.1 mm in female and 11.4 mm in juvenile male); head width (HW 9.72 mm in female and 7.6 mm in juvenile male); tail tip pointed; number of annuli: total annuli 375 in female and 365 in juvenile male, annuli interrupted by vent 6 in female and 5 in juvenile male, post-vent annuli 4 in both sexes. Colouration in life: dorsal surface of head, body and tail lilac; lateral stripe bright yellow, broad, interrupted posteriorly, extending from behind tentacle on upper jaw to posterior end of vent; ventral surface bright lilac (Fig. 3).

#### Agamidae

BABFE4F3-9EA4-5B71-810C-7E19CC61CE39

#### Acanthosaura lepidogaster

(Cuvier, 1829)

636B831A-FE59-5383-A9FA-AA64F71FB52A

##### Native status

Not threatened

##### Conservation status

LC (IUCN 2025)

##### Distribution

Vietnam (Ba Vi: Nguyen et al. (2009)), China, Myanmar, Laos, Thailand and Cambodia (Nguyen et al. 2009).

##### Notes

Previously recorded from BVNP by Nguyen et al. (2009), observed during the present surveys.

#### Calotes emma

Gray, 1845

E5717B92-3E72-5422-86CF-F7D857E4A1D5

##### Materials

- **Type status:** Other material. **Occurrence:** catalogNumber: VNUF R.2024.102; individualCount: 1; sex: male; lifeStage: adult; occurrenceID: 54770819-F590-5AB3-B813-FB023E07FD2E; **Taxon:** genus: Calotes; specificEpithet: *emma*; **Location:** country: Vietnam; stateProvince: Phu Tho; municipality: Ky Son; locality: Ba Vi National Park; verbatimElevation: 193 m; verbatimCoordinates: 20 56.032N, 105 26.621E; verbatimCoordinateSystem: degrees decimal minutes; **Event:** eventDate: 29 July 2024; **Record Level:** institutionCode: VNUF
- **Type status:** Other material. **Occurrence:** catalogNumber: VNUF R.2024.102; individualCount: 1; sex: female; lifeStage: adult; occurrenceID: 5371241B-8397-51B7-AD14-B395D19EB979; **Taxon:** genus: Calotes; specificEpithet: *emma*; **Location:** country: Vietnam; stateProvince: Phu Tho; municipality: Ky Son; locality: Ba Vi National Park; verbatimElevation: 170 m; verbatimCoordinates: 20 55.914N, 105 26.615E; verbatimCoordinateSystem: degrees decimal minutes; **Event:** eventDate: 31 July 2024; **Record Level:** institutionCode: VNUF

##### Native status

Not threatened

##### Conservation status

LC (IUCN 2025)

##### Distribution

In Vietnam, this species is known from Cao Bang and Bac Kan Provinces in the north, southwards to Ba Ria-Vung Tau and Kon Tum Provinces in central Vietnam. Elsewhere, this species has been reported from India, China, Myanmar, Laos, Thailand, Cambodia and Malaysia (Nguyen et al. 2009). First record in BVNP.

##### Notes

Both specimens were found on tree branches between 23:30 h and 00:24 h. The surrounding habitats were nature forest, streams and cliffs. The relative temperature was 25–27°C and humidity was 90–93%.

##### Diagnosis

**Figure 4.**
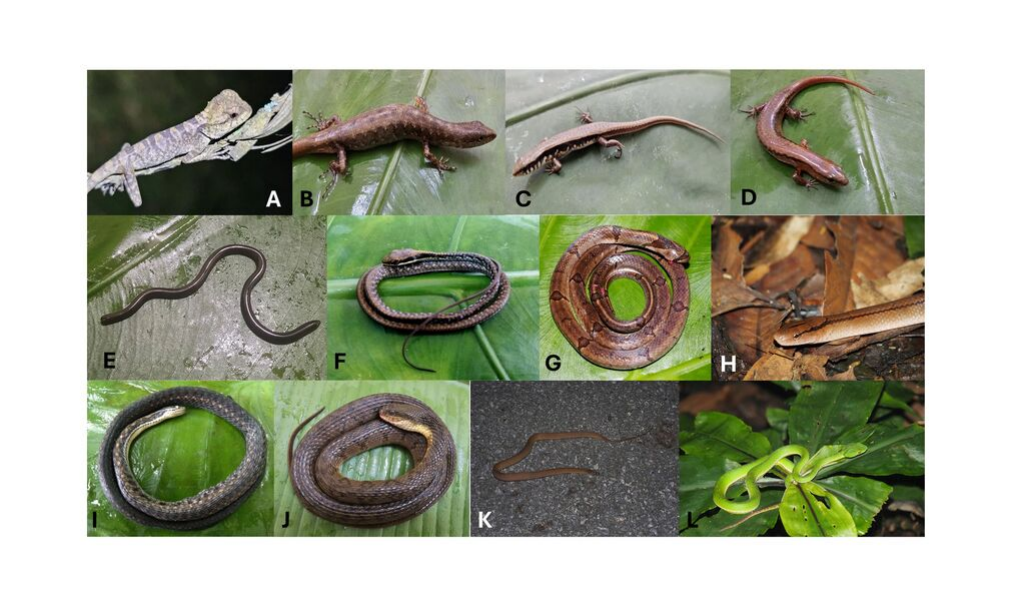
Twelve newly-recorded reptile species from BVNP. **A** *Calotes emma*; **B** *Sphenomorphus cryptotis*; **C** *Sphenomorphus indicus*; **D** Sphenomorphus cf. tonkinensis; **E** Indotyphlops cf. braminus; **F** *Dendrelaphis ngansonensis*; **G** *Oligodon chinensis*; **H** *Oreocryptophis porphyraceus*; **I** *Hebius boulengeri*; **J** *Trimerodytes percarinatus*; **K** Achalinus cf. juliani; **L** *Trimeresurus stejnegeri*. Photographs by Dinh T.S. & K. Nishikawa.

Morphological characters of the specimen from BVNP agreed well with descriptions of Taylor (1963) and Nguyen et al. (2016): SVL 90.7 mm in male and 90.1 mm in female, TaL 262.0 mm in male and 288.0 mm in female; head length about 1.5 times its width HL 26.6 mm in male and 27.6 mm in female, HW 18.0 mm in male and 17.0 mm in the adult female; supralabials 8/10 in male and 11/11 in female; infralabials 9/9 in male and 10/10 in female; scale rows at mid-body 56 in male and 54 in female; scales in a line from mental to the front of cloacal slit 83 in the male and 91 in the female; subdigital lamellae under fourth finger 23, under fourth toe 27–28. Colouration in life: Dorsum olive-brown with dark brown dorsal bars or transverse spots; radiating dark lines from eye; ventral greyish-cream (Fig. 4).

#### Draco maculatus

(Gray, 1845)

695985EE-DD95-52EE-8923-7DD78C65EBE0

##### Native status

Not threatened

##### Conservation status

LC (IUCN 2025)

##### Distribution

Vietnam (Ba Vi: Nguyen et al. (2009)), India, China, Myanmar, Laos, Thailand, Cambodia and Malaysia (Nguyen et al. 2009).

##### Notes

Previously recorded from BVNP by Nguyen et al. (2009), not observed during the present surveys.

#### Pseudocalotes brevipes

(Werner, 1904)

650A2EB9-0E76-5880-BD98-A3CD33BAC403

##### Native status

Not threatened

##### Conservation status

LC (IUCN 2025)

##### Distribution

Vietnam (Ba Vi: Nguyen et al. (2009)) and China (Nguyen et al. 2009).

##### Notes

Previously recorded from BVNP by Nguyen et al. (2009), not observed during the present surveys.

#### Gekkonidae

0C8FFFCD-F665-54FB-A5E1-2EFC53C735E4

#### Gekko palmatus

Boulenger, 1907

B703EF29-A808-5464-8D3C-C199995CED2D

##### Native status

Not threatened

##### Conservation status

LC (IUCN 2025)

##### Distribution

Vietnam (Ba Vi: Luu et al. (2020)) and China (Luu et al. 2020).

##### Notes

Previously recorded from BVNP by Luu et al. (2020), observed during the present surveys.

#### Gekko reevesii

(Gray, 1831)

BA8EA3F6-4F33-5712-AA2C-343EB86C3894

##### Native status

VU (VNREDLIST 2024)

##### Conservation status

LC (IUCN 2025)

##### Distribution

Vietnam (northern and southwards to Quang Binh Province) and China by Rösler et al. (2011).

##### Notes

Previously recorded from BVNP by Luu et al. (2020), observed during the present surveys.

#### Goniurosaurus lichtenfelderi

(Mocquard, 1897)

4E141491-90B5-54BD-A576-65FEEFA1EECC

##### Native status

VU (VNREDLIST 2024)

##### Conservation status

VU (IUCN 2025)

##### Distribution

Vietnam (Cao Bang, Lang Son, Ba Vi, Quang Ninh, Bac Giang, Hai Hung Provinces, Orlov et al. (2020)) and China (Zhu et al. 2020).

##### Notes

Previously recorded from BVNP by Nguyen et al. (2009), not observed during the present surveys.

#### Hemidactylus frenatus

Duméril & Bibron, 1836

A81A04D4-304E-5E03-85BD-B959579C9B28

##### Native status

Not threatened

##### Conservation status

LC (IUCN 2025)

##### Distribution

Vietnam (entire country: Nguyen et al. (2009)), worldwide in tropical and subtropical regions (Nguyen et al. 2009).

##### Notes

Previously recorded from BVNP by Nguyen et al. (2009), observed during the present surveys.

#### Scincidae

30A95699-1427-5EA8-9A35-FD871738451C

#### Eutropis longicaudata

(Hallowell, 1857)

77CCB436-1257-561B-BA12-1A2011A1F213

##### Native status

Not threatened

##### Conservation status

LC (IUCN 2025)

##### Distribution

Vietnam (entire country: Nguyen et al. (2009)), China, Taiwan, Laos, Thailand, Cambodia and Malaysia (Nguyen et al. 2009).

##### Notes

Previously recorded from BVNP by Nguyen et al. (2009), observed during the present surveys.

#### Plestiodon tamdaoensis

(Bourret, 1937)

04B3C601-A1FA-5C60-A40F-1F69CC6CF693

##### Native status

Not threatened

##### Conservation status

LC (IUCN 2025)

##### Distribution

Vietnam (BVNP: Pham et al. (2025)) and China (Pham et al. 2025).

##### Notes

Previously recorded from BVNP by Pham et al. (2025), not observed during the present surveys.

#### Scincella devorator

Darevsky, Orlov & Cuc, 2004

CECDFD33-14BB-57A1-810C-A7FBAA9E23B8

##### Native status

NT (VNREDLIST 2024)

##### Conservation status

DD (IUCN 2025)

##### Distribution

This species is only known in Vietnam (Pham et al. 2025). This species has been reported in BVNP by Pham et al. (2025).

##### Notes

Previously recorded from BVNP by Pham et al. (2025), not observed during the present surveys.

#### Sphenomorphus cryptotis

Darevsky, Orlov & Cuc, 2004

E72888E6-C3FB-503F-BD4E-890C0B5C7FED

##### Materials

- **Type status:** Other material. **Occurrence:** catalogNumber: VNUF R.2024.78; individualCount: 1; sex: male; lifeStage: adult; occurrenceID: E5812193-0936-570F-9589-AC36DE8919F6; **Taxon:** genus: Sphenomorphus; specificEpithet: *cryptotis*; **Location:** country: Vietnam; stateProvince: Hanoi; municipality: Ba Vi; locality: Ba Vi National Park; verbatimElevation: 450 m; verbatimCoordinates: 21 04.927N, 105 22.027E; verbatimCoordinateSystem: degrees decimal minutes; **Event:** eventDate: 21 July 2024; **Record Level:** institutionCode: VNUF
- **Type status:** Other material. **Occurrence:** catalogNumber: VNUF R.2024.79; individualCount: 1; sex: female; lifeStage: adult; occurrenceID: A938BEBB-DE80-5740-B968-ED380F669BB8; **Taxon:** genus: Sphenomorphus; specificEpithet: *cryptotis*; **Location:** country: Vietnam; stateProvince: Hanoi; municipality: Ba Vi; locality: Ba Vi National Park; verbatimElevation: 450 m; verbatimCoordinates: 21 04.927N, 105 22.027E; verbatimCoordinateSystem: degrees decimal minutes; **Event:** eventDate: 21 July 2024; **Record Level:** institutionCode: VNUF

##### Native status

Not threatened

##### Conservation status

LC (IUCN 2025)

##### Distribution

In Vietnam, this species has been reported from Dien Bien to Nghe An Provinces (Luong et al. 2019). Elsewhere, this species has been reported from China (Qi et al. 2022). First record in BVNP.

##### Notes

Both specimens were found on tree leaves in Ngoc Hoa Stream between 21:00 h and 0:31 h. The surrounding habitats were secondary forest, streams, bamboo forest and banana forest. The relative temperature was 24–26°C and humidity was 91–96%.

##### Diagnosis

Morphological characters of the specimen from BVNP agreed well with descriptions of Darevsky et al. (2004), Hecht et al. (2013) and Qi et al. (2022): SVL 67.0 mm in male and 65.5 mm in female; TaL 126.6 mm in male and 96.2 mm in female; head moderately sized, longer than wide (HL 12.7 mm in male and 12.1 mm in female, HW 9.4 mm in male and 8.0 mm in female), subtriangular, distinct from neck; rostral wider than high; supranasals absent; frontonasal wider than long, in contact with nasal, rostral, anterior loreal and prefrontal; loreals 2; supraoculars 4; primary temporal single; secondary temporals 2, upper very large and overlapped by lower one; supralabials 7, the fifth and sixth below the eye; infralabials 7; post mental undivided; mid-body scales in 34 rows; paravertebral scales 68 rows in male and 71 rows in female; ventrals scales 75 rows in male and 81 rows in female, precloacals 2, enlarged; subdigital lamellae on fourth finger 12; subdigital lamellae on fourth toe 16 in female, 19/11 in male. Colouration in life: dorsum and tail base yellowish-brown with a vertebral row of large black spots; numerous indistinct white spots on the labials; lateral zone with a distinct dark stripe from behind the eye to tail base, with white spots; venter and underside anterior part of tail white, posterior part of tail yellowish-brown (Fig. 4).

#### Sphenomorphus indicus

(Gray, 1853)

3298965F-927A-5D6A-8E32-F033F53D8EE9

##### Materials

- **Type status:** Other material. **Occurrence:** catalogNumber: VNUF R.2024.11; individualCount: 1; sex: female; lifeStage: juvenile; occurrenceID: E993AA7A-AFAC-5960-ACE8-22F2B3F56EEA; **Taxon:** genus: Sphenomorphus; specificEpithet: *indicus*; **Location:** country: Vietnam; stateProvince: Hanoi; municipality: Ba Vi; locality: Ba Vi National Park; verbatimElevation: 378 m; verbatimCoordinates: 21 05.029N, 105 22.807E; verbatimCoordinateSystem: degrees decimal minutes; **Event:** eventDate: 10 July 2024; **Record Level:** institutionCode: VNUF
- **Type status:** Other material. **Occurrence:** catalogNumber: VNUF R.2024.12; individualCount: 1; sex: female; lifeStage: juvenile; occurrenceID: 71EBAA1F-AD58-5647-9AB1-B545C7199374; **Taxon:** genus: Sphenomorphus; specificEpithet: *indicus*; **Location:** country: Vietnam; stateProvince: Hanoi; municipality: Ba Vi; locality: Ba Vi National Park; verbatimElevation: 378 m; verbatimCoordinates: 21 05.029N, 105 22.807E; verbatimCoordinateSystem: degrees decimal minutes; **Event:** eventDate: 10 July 2024; **Record Level:** institutionCode: VNUF

##### Native status

Not threatened

##### Conservation status

LC (IUCN 2025)

##### Distribution

This species has a wide range from northern to southern provinces of Vietnam. Elsewhere, this species has been reported from India, Bhutan, China, Taiwan, Myanmar, Laos, Thailand, Cambodia, Malaysia and Indonesia (Nguyen et al. 2009). First record in BVNP.

##### Notes

Both specimens were found on the grassland at 0:30 h. The relative temperature was 25°C and humidity was 92%.

##### Diagnosis

Morphological characters of the specimens from BVNP agreed well with the descriptions of Hecht et al. (2013) and Nemes et al. (2013): SVL 40.4–40.6 mm; TaL 65.1–67.6 mm; head longer than wide (HL 7.8–8.6 mm, HW 6.6–6.7 mm); loreals 2; supraciliaries 8; supraoculars 4; primary temporals 2; secondary temporals 2; supralabials 7, the fifth and sixth below the eye, separated from it by the row of small scales; infralabials 7; post mental undivided; mid-body scales in 36 rows; paravertebral scales 65–67 rows; ventrals scales 65–67 rows, precloacals 2, enlarged; subdigital lamellae on fourth finger 9–11; subdigital lamellae on fourth toe 15. Colouration in life: dorsum and tail base bronze-brown with irregular black dots; light dorsolateral stripe present on neck and shoulder; upper lateral zone with a dark stripe, from behind eye to tail base, paler on distal tail, the lower margin of dark stripe with some broken light spots; ventral surface whitish (Fig. 4).

#### Sphenomorphus cf. tonkinensis

Nguyen, Schmitz, Nguyen, Orlov, Böhme & Ziegler, 2011

6686E97F-2370-5040-92FC-D4C18C454CBA

##### Materials

- **Type status:** Other material. **Occurrence:** catalogNumber: IB A.2024.043; individualCount: 1; sex: female; lifeStage: adult; occurrenceID: 998262CC-AD91-5D0D-A31F-8D34690A1A29; **Taxon:** genus: Sphenomorphus; specificEpithet: cf. *tonkinensis*; **Location:** country: Vietnam; stateProvince: Hanoi; municipality: Ba Vi; locality: Ba Vi National Park; verbatimElevation: 722 m; verbatimCoordinates: 21 04.581N, 105 22.009E; verbatimCoordinateSystem: degrees decimal minutes; **Event:** eventDate: 14 July 2024; **Record Level:** institutionCode: IB

##### Native status

Not threatened

##### Conservation status

LC (IUCN 2025)

##### Distribution

In Vietnam, this species has been recorded from Vinh Phuc, Quang Ninh and Hai Phong Provinces. Elsewhere, this species has been reported from China (Nguyen et al. 2011a). First record in BVNP.

##### Notes

The specimen was found on the ground at 23:30 h in the French Camping area. The surrounding habitats were secondary forest, streams, bamboo forest and banana forest. The relative temperature was 25°C and the humidity was 89%.

##### Diagnosis

Morphological characters of the specimens from BVNP almost agreed with the descriptions of Nguyen et al. (2011a) and Gawor et al. (2016): SVL 49.0 mm; TaL 41.4 mm; head longer than wide (HL 7.7 mm, HW 5.71 mm); loreals 2; supraciliaries 7; supraoculars 4; primary temporals 2; secondary temporals 2; supralabials 7, the fifth and sixth below the eye, separated from it by the row of small scales; infralabials 7; post mental undivided; mid-body scales in 36 rows; paravertebral scales 70 rows; ventral scales 65 rows, precloacals 2, enlarged; subdigital lamellae on fourth finger 7/8; subdigital lamellae on fourth toe 13/14. Colouration in life: dorsum and tail base bronze-brown with black, discontinuous vertebral line reaching to first third of tail, tail orange brown, ventral surface cream (Fig. 4).

#### Tropidophorus baviensis

Bourret, 1939

C710B3CA-F3B1-5BFC-9418-7B0B73A2423A

##### Native status

Not threatened

##### Conservation status

LC (IUCN 2025)

##### Distribution

Vietnam (Ba Vi: Nguyen et al. (2009)) and Laos (Okabe et al. 2020).

##### Notes

Previously recorded from BVNP by Nguyen et al. (2009), observed during the present surveys.

#### Tropidophorus hainanus

Smith, 1923

AE8847C8-A6C4-57EA-A00C-2F844E85894B

##### Native status

Not threatened

##### Conservation status

LC (IUCN 2025)

##### Distribution

Vietnam (Ba Vi: Nguyen et al. (2009)) and China (Nguyen et al. 2009).

##### Notes

Previously recorded from BVNP by Nguyen et al. (2009), observed during the present surveys.

#### Anguidae

5C4213E0-8CF0-5FC1-BDB8-A4BE4DA84628

#### Dopasia harti

(Boulenger, 1899)

91D55DD1-AD5F-5E75-83E0-0C83E57EC8FF

##### Native status

Not threatened

##### Conservation status

LC (IUCN 2025)

##### Distribution

Vietnam (Ba Vi: Nguyen et al. (2009)), China, Taiwan and Laos (Nguyen et al. 2009).

##### Notes

Previously recorded from BVNP by Nguyen et al. (2009), not observed during the present surveys.

#### Dopasia ludovici

(Mocquard, 1905)

1A7244D2-28B4-599D-A47E-C5ABEDEF2AA9

##### Native status

Not threatened

##### Conservation status

LC (IUCN 2025)

##### Distribution

Vietnam (Ba Vi: Nguyen et al. (2011b)) and China (Nguyen et al. 2011b).

##### Notes

Previously recorded from BVNP by Nguyen et al. (2011b), observed during the present surveys.

#### Typhlopidae

49719BFB-E5F8-5A7D-B27D-2292C083C53A

#### Indotyphlops cf. braminus

(Daudin, 1803)

5C8A141D-42CA-5E86-90EA-2688133DDAE0

##### Materials

- **Type status:** Other material. **Occurrence:** catalogNumber: IB A.2024.103; individualCount: 1; lifeStage: juvenile; occurrenceID: C053DFDD-F86F-5A66-B825-B93954BA5EF5; **Taxon:** genus: Indotyphlops; specificEpithet: cf. *braminus*; **Location:** country: Vietnam; stateProvince: Phu Tho; municipality: Ky Son; locality: Ba Vi National Park; verbatimElevation: 200 m; verbatimCoordinates: 20 56.015N, 105 26.618E; verbatimCoordinateSystem: degrees decimal minutes; **Event:** eventDate: 29 July 2024; **Record Level:** institutionCode: IB
- **Type status:** Other material. **Occurrence:** catalogNumber: IB A.2024.114; individualCount: 1; lifeStage: juvenile; occurrenceID: 8A92DE12-180B-5E35-948E-AF6C81A3CECB; **Taxon:** genus: Indotyphlops; specificEpithet: cf. *braminus*; **Location:** country: Vietnam; stateProvince: Phu Tho; municipality: Ky Son; locality: Ba Vi National Park; verbatimElevation: 200 m; verbatimCoordinates: 20 56.015N, 105 26.618E; verbatimCoordinateSystem: degrees decimal minutes; **Event:** eventDate: 29 July 2024; **Record Level:** institutionCode: IB

##### Native status

Not threatened

##### Conservation status

LC (IUCN 2025)

##### Distribution

This species has a wide range from northern to southern of Vietnam (Hecht et al. 2013). Elsewhere, this species has been reported from Africa, Asia, Oceania and America (Luong et al. 2025). First record in BVNP.

##### Notes

The specimens were found at 1:54 h under a rock. The surrounding habitats were evergreen forest and streams. The relative temperature was 27°C and humidity was 94%.

##### Diagnosis

Morphological characters of the specimens from BVNP almost agreed with the descriptions of Vassilieva et al. (2016): SVL 68.0–84.0 mm; TaL 2.0 mm in both specimens; body thin, cylindrical, vermiform; head small, but indistinct from neck; snout short and rounded; eyes very small, but distinct; mid-body scale rows 20, scales smooth, glossy; total mid-dorsal scales 317–328; subcaudals 9–12. Colouration in life: dorsal dark brown, undersides paler (Fig. 4).

#### Colubridae

4BE2E2D5-E078-5599-9E44-8FDFCD59482B

#### Ahaetulla prasina

(Boie, 1827)

24B44A01-60DF-508E-B93E-EF7796AC2E78

##### Native status

Not threatened

##### Conservation status

LC (IUCN 2025)

##### Distribution

Vietnam (Ba Vi: Nguyen et al. (2009)), India, Bhutan, Myanmar, Cambodia, Laos, Vietnam, Malaysia, China, Indonesia and Philippines (Nguyen et al. 2009).

##### Notes

Previously recorded from BVNP by Nguyen et al. (2009), observed during the present surveys.

#### Boiga guangxiensis

Wen, 1998

B583C882-92BD-5C24-8B03-0C6242E00633

##### Native status

Not threatened

##### Conservation status

LC (IUCN 2025)

##### Distribution

Vietnam (Ba Vi: Luu et al. (2020)), China, Cambodia and Laos (Luu et al. 2020).

##### Notes

Previously recorded from BVNP by Luu et al. (2020), observed during the present surveys.

#### Boiga multomaculata

(Boie, 1827)

3580587A-CCCE-573A-8045-96407B9C2AF3

##### Native status

Not threatened

##### Conservation status

LC (IUCN 2025)

##### Distribution

Vietnam (Ba Vi: Nguyen et al. (2009)), India, Myanmar, Cambodia, Laos, Vietnam, Malaysia, China, Indonesia and Singapore (Nguyen et al. 2009).

##### Notes

Previously recorded from BVNP by Nguyen et al. (2009), observed during the present surveys.

#### Calamaria pavimentata

Duméril, Bibron & Duméril, 1854

345E674A-81DC-5CD7-AEEE-807581008EE4

##### Native status

Not threatened

##### Conservation status

LC (IUCN 2025)

##### Distribution

Vietnam (Ba Vi: Nguyen et al. (2009)), China, Malaysia, Thailand, Laos, Cambodia, Myanmar and Indonesia (Nguyen et al. 2009).

##### Notes

Previously recorded from BVNP by Nguyen et al. (2009), observed during the present surveys.

#### Calamaria septentrionalis

Boulenger, 1890

18C7E647-A6AD-597A-985E-33CB02600802

##### Native status

Not threatened

##### Conservation status

LC (IUCN 2025)

##### Distribution

Vietnam (Ba Vi: Nguyen et al. (2009)) and China (Nguyen et al. 2009).

##### Notes

Previously recorded from BVNP by Nguyen et al. (2009), observed during the present surveys.

#### Coelognathus radiatus

(Boie, 1827)

F9BE779F-9E98-59C5-AB16-32A4E13A8BD4

##### Native status

NT (VNREDLIST 2024)

##### Conservation status

LC (IUCN 2025)

##### Distribution

Vietnam (entire country: Nguyen et al. (2009)), Indonesia, Malaysia, Myanmar, Thailand, Laos, Cambodia, India, Banglagesh, Nepal and China (Nguyen et al. 2009).

##### Notes

Previously recorded from BVNP by Nguyen et al. (2009), observed during the present surveys.

#### Dendrelaphis ngansonensis

(Bourret, 1935)

CB78CA96-51A7-5BF6-9C2B-358F032075EF

##### Materials

- **Type status:** Other material. **Occurrence:** catalogNumber: VNUF R.2021.02; individualCount: 1; sex: female; lifeStage: juvenile; occurrenceID: 58F567DF-7824-5DA3-9C66-04295BBD7217; **Taxon:** genus: Dendrelaphis; specificEpithet: *ngansonensis*; scientificNameAuthorship: Bourret, 1935; **Location:** country: Vietnam; stateProvince: Hanoi; municipality: Ba Vi; locality: Ba Vi National Park; verbatimElevation: 435 m; verbatimCoordinates: 21 08.778N, 105 37.141E; verbatimCoordinateSystem: degrees decimal minutes; **Event:** eventDate: 7 April 2021; **Record Level:** institutionCode: VNUF

##### Native status

Not threatened

##### Conservation status

LC (IUCN 2025)

##### Distribution

In Vietnam, this species is known from Ha Giang Province in the north, southwards to Quang Nam Province (Nguyen et al. 2009). Elsewhere, this species has been reported from China, Laos, Thailand and Cambodia (Uetz et al. 2026). First record in BVNP.

##### Notes

The specimen was found in the morning on the road. The surrounding habitat was pine forest. The relative temperature was 28°C and humidity was 80%.

##### Diagnosis

Morphological characters of the specimens from BVNP agreed well with descriptions of Nemes et al. (2013) and Neang et al. (2015): SVL 250.0 mm, TaL 123.0 mm; head longer than wide (HL 12.4 mm, HW 7.2 mm), distinct from neck; pupil round; internasal shorter than prefrontal; loreal 1/1, not touching the eye; preocular 1/1; postoculars 2/2; anterior temporals 2/2, posterior temporals 2/2; supralabials 9/9, fourth to sixth in contact with the eye, seven largest; infralabials 10/10, first to fifth bordering chin shields; dorsal scale rows 15–15–11, smooth; ventrals 181; cloacal divided; subcaudals 141, divided. Colouration in life: dorsum bronze-brown; interstitial skin black and white; dorsal head bronze-brown; a black stripe present along the side of the head, strongly marked on the temple and extending to the neck; supralabials yellow; cream below (Fig. 4).

#### Gonyosoma boulengeri

(Mocquard, 1897)

938DAD5B-8C59-566C-9163-18894652844D

##### Native status

Not threatened

##### Conservation status

LC (IUCN 2025)

##### Distribution

Vietnam (Ba Vi: Nguyen et al. (2009)) and China (Nguyen et al. 2009).

##### Notes

Previously recorded from BVNP by Nguyen et al. (2009), observed during the present surveys.

#### Gonyosoma coeruleum

Liu, Hou, Lwin, Wang & Rao, 2021

AB55C855-6FD5-5535-82F4-A22B2837AA35

##### Native status

Not threatened

##### Conservation status

Not evaluated

##### Distribution

Vietnam (Ba Vi: Luu et al. (2020)), India, China, Myanmar, Laos, Thailand and Malaysia (Luu et al. 2020).

##### Notes

Previously recorded from BVNP by Luu et al. (2020), not observed during the present surveys.

#### Lycodon futsingensis

(Pope, 1928)

AD2EB3B0-28DF-5A83-9CF7-C4D0BA0A6445

##### Native status

Not threatened

##### Conservation status

LC (IUCN 2025)

##### Distribution

Vietnam (Ba Vi: Luu et al. (2020)), China, Laos, Japan, Taiwan, and Myanmar (Luu et al. 2020).

##### Notes

Previously recorded from BVNP by Luu et al. (2020), observed during the present surveys.

#### Lycodon flavozonatus

(Pope, 1928)

59960F04-50D8-572D-AB5A-30EF129EB727

##### Native status

Not threatened

##### Conservation status

LC (IUCN 2025)

##### Distribution

Vietnam (Ba Vi: Nguyen et al. (2009)), China and Laos (Nguyen et al. 2009).

##### Notes

Previously recorded from BVNP by Nguyen et al. (2009), observed during the present surveys.

#### Lycodon ruhstrati abditus

Vogel, David, Pauwels, Sumontha, Norval, Hendrix, Vu & Ziegler, 2009

6F73BA51-F134-5000-A809-FFD669C3C167

##### Native status

Not threatened

##### Conservation status

Not evaluated

##### Distribution

Vietnam (Ba Vi: Luu et al. (2020)), China and Laos (Luu et al. 2020).

##### Notes

Previously recorded from BVNP by Luu et al. (2020), not observed during the present surveys.

#### Lycodon neomaculatus

(Nguyen, Lee, Pauwels, Kennedy-Gold, Poyarkov, David & Vogel, 2024)

48A68D9E-711E-5D77-9724-2DFAB8E15FEB

##### Native status

Not threatened

##### Conservation status

Not evaluated

##### Distribution

Vietnam (Ba Vi: Nguyen et al. (2009)), China, Myanmar, Laos, Cambodia and Thailand (Nguyen et al. 2024).

##### Notes

Previously recorded from BVNP by Nguyen et al. (2009), observed during the present surveys.

#### Oligodon cinereus

(Günther, 1864)

33FAD348-E2C3-59BF-A8E6-9BBCBAB2F3F6

##### Native status

Not threatened

##### Conservation status

LC (IUCN 2025)

##### Distribution

Vietnam (Ba Vi: Nguyen et al. (2009)), India, Bangladesh, China, Myanmar, Laos, Thailand, Cambodia and Malaysia (Nguyen et al. 2009).

##### Notes

Previously recorded from BVNP by Nguyen et al. (2009), not observed during the present surveys.

#### Oligodon chinensis

(Günther, 1888)

754E0195-64ED-53A8-BD0E-112C6C637E40

##### Materials

- **Type status:** Other material. **Occurrence:** catalogNumber: IB.A.2025.078; individualCount: 1; sex: male; lifeStage: juvenile; occurrenceID: C6D86CB9-7531-51DC-BE9B-BF8AE44C1A3E; **Taxon:** genus: Oligodon; specificEpithet: *chinensis*; **Location:** country: Vietnam; stateProvince: Hanoi; municipality: Ba Vi; locality: Ba Vi National Park; verbatimElevation: 600 m; verbatimCoordinates: 21 04.576N, 105 22.137E; verbatimCoordinateSystem: degrees decimal minutes; **Event:** eventDate: 4 July 2025; **Record Level:** institutionCode: IB

##### Native status

Not threatened

##### Conservation status

LC (IUCN 2025)

##### Distribution

In Vietnam, this is a widespread species known from Lao Cai, Lang Son, Ha Giang and Son La in the north, southwards to Quang Binh and Gia Lai Provinces. Elsewhere, this species has been reported from China (Nguyen et al. 2009, Pham et al. 2014). First record in BVNP.

##### Notes

The specimen was found at 21:00 h on the main road. The relative temperature was 28°C and humidity was 80%.

##### Diagnosis

Morphological characters of the specimens from BVNP agreed well with descriptions of Hecht et al. (2013), Pham et al. (2014) and Gawor et al. (2016): SVL 416.0 mm; TaL 98.0 mm; Body robust, subcylindrical; head short, longer than wide (HL 12.6 mm, HW 9.3 mm), indistinct from neck; loreal 1/1, square in shape, not touching the eye; preocular 2/2; postoculars 2/2; anterior temporals 2/2, posterior temporals 1/2; supralabials 8/7, fourth and fifth in contact with the eye, sixth largest; infralabials 8/8, first to fourth bordering chin shields; dorsal scale rows 17–17–15, smooth; ventrals 184; cloacal single; subcaudals 60, divided. Colouration in life: dorsal surface brown with 14 distinct reddish-brown spots on body and 3 on tail, a black band running from one eye to the other, V-marking present behind head. Ventral face white with squarish spots on the outer margins of the ventrals (Fig. 4).

#### Oligodon eberhardti

Pellegrin, 1910

9180F7DE-9005-54FC-AE2C-F596798C71B7

##### Native status

Not threatened

##### Conservation status

LC (IUCN 2025)

##### Distribution

Vietnam (Ba Vi: Nguyen et al. (2009)), China, Myanmar, Laos and Cambodia (Nguyen et al. 2009).

##### Notes

Previously recorded from BVNP by Nguyen et al. (2009), not observed during the present surveys.

#### Oligodon fasciolatus

(Günther, 1864)

4D459184-4DED-57D8-99E1-3D5E36FBBC52

##### Native status

Not threatened

##### Conservation status

LC (IUCN 2025)

##### Distribution

Vietnam (Ba Vi: Nguyen et al. (2009)), Myanmar, Thailand, Cambodia, Laos, Vietnam and China (Uetz et al. 2026).

##### Notes

Previously recorded from BVNP by Nguyen et al. (2009), not observed during the present surveys.

#### Oreocryptophis porphyraceus

(Cantor, 1839)

962B7DDC-7F18-501E-A22F-F888B784D375

##### Materials

- **Type status:** Other material. **Occurrence:** catalogNumber: IB A.2025.040; individualCount: 1; sex: male; lifeStage: adult; occurrenceID: C053D7C6-32C9-51E2-B4C3-6CE13AA92B1B; **Taxon:** scientificNameID: *Oreocryptophis porphyraceus*; scientificName: *Oreocryptophis porphyraceus*; class: Reptilia; order: Squamata; family: Colubridae; genus: Oreocryptophis; specificEpithet: *porphyraceus*; scientificNameAuthorship: Cantor, 1839; **Location:** country: Vietnam; stateProvince: Hanoi; municipality: Ba Vi; locality: Ba Vi National Park; verbatimElevation: 1000 m; verbatimCoordinates: 21 04.543N, 105 22.619E; verbatimCoordinateSystem: degrees decimal minutes; **Event:** eventDate: 17 June 2025; **Record Level:** institutionCode: IB

##### Native status

NT (VNREDLIST 2024)

##### Conservation status

LC (IUCN 2025)

##### Distribution

In Vietnam, this species is known from Lao Cai, Ha Giang and Yen Bai Provinces in the north, southwards to Quang Binh Province in central Vietnam. Elsewhere, this species has been reported from India, Nepal, China, Taiwan, Myanmar, Laos, Thailand, Cambodia, Malaysia and Indonesia (Nguyen et al. 2009). First record in BVNP.

##### Notes

The specimen was found on the ground at 22:00 h in the French Era Political Prison (ruins). The surrounding habitats were evergreen forest, streams and cliffs. The relative temperature was 28°C and humidity was 78%.

##### Diagnosis

Morphological characters of the specimens from BVNP agreed well with descriptions of Ziegler et al. (2014) and Le et al. (2018): SVL 650.0 mm, Tal 155.0 mm; body slender; head elongated, longer than wide (HL 23.4 mm, HW 11.0 mm), slightly distinct from neck; loreal 1/1, square in shape, not touching the eye; preocular 1/1; postoculars 2/2; anterior temporals 1/1, posterior temporals 2/2; supralabials 8/8, fourth and fifth in contact with the eye, seventh largest; infralabials 10/10, first to fourth bordering chin shields; dorsal scale rows 19–19–17, smooth; ventrals 207; cloacal divided; subcaudals 78, divided. Colouration in life: dorsal surface of head, body and tail red, two black stripes on posterior body and tail and another on dorsal head; venter and lower surface of tail cream (Fig. 4).

#### Ptyas korros

(Schlegel, 1837)

BAF983AB-F84C-528B-BCF4-501FE829EEBE

##### Native status

VU (VNREDLIST 2024)

##### Conservation status

NT (IUCN 2025)

##### Distribution

Vietnam (Ba Vi: Nguyen et al. (2009)), India, Bangladesh, China, Taiwan, Myanmar, Laos, Thailand, Cambodia, Malaysia, Singapore and Indonesia (Nguyen et al. 2009).

##### Notes

Previously recorded from BVNP by Nguyen et al. (2009), observed during the present surveys.

#### Ptyas major

(Günther, 1858)

0FB926E6-C239-55CE-9543-8CD06BD4D2D4

##### Native status

Not threatened

##### Conservation status

LC (IUCN 2025)

##### Distribution

Vietnam (Ba Vi: Nguyen et al. (2009)), China, Taiwan and Laos (Nguyen et al. 2009).

##### Notes

Previously recorded from BVNP by Nguyen et al. (2009), not observed during the present surveys.

#### Ptyas multicincta

(Roux, 1907)

D9AFC1E6-5E70-56C2-8F11-A6DC2A658B64

##### Native status

Not threatened

##### Conservation status

LC (IUCN 2025)

##### Distribution

Vietnam (Ba Vi: Nguyen et al. (2009)), China and Laos (Nguyen et al. 2009).

##### Notes

Previously recorded from BVNP by Nguyen et al. (2009), observed during the present surveys.

#### Ptyas nigromarginata

(Blyth, 1855)

8DB0791F-2096-5473-8AE2-A304ED0E810F

##### Native status

Not threatened

##### Conservation status

LC (IUCN 2025)

##### Distribution

Vietnam (Ba Vi: Nguyen et al. (2009)), India, Nepal, China, Myanmar and Laos (Nguyen et al. 2009).

##### Notes

Previously recorded from BVNP by Nguyen et al. (2009), not observed during the present surveys.

#### Homalopsidae

900EC4C9-2E5F-58DB-A98D-D2FB1AAA6B62

#### Hypsiscopus murphyi

Bernstein, Voris, Stuart, Phimmachak, Seateun, Sivongxay, Neang, Karns, Andrews, Osterhage, Phipps & Ruane, 2022

7B535380-EE91-5028-A448-CCA16777C2EC

##### Native status

Not threatened

##### Conservation status

Not evaluated

##### Distribution

Vietnam (entire country: Nguyen et al. (2009)), Cambodia, China, Laos, and Thailand (Bernstein et al. 2022).

##### Notes

Previously recorded from BVNP by Nguyen et al. (2009), not observed during the present surveys.

#### Natricidae

376E051B-6DA0-50A1-8697-DC1BE2183C5D

#### Fowlea flavipunctata

(Hallowell, 1860)

EFFCD2BD-82D4-50DA-B7D8-473A2E160AA7

##### Native status

Not threatened

##### Conservation status

LC (IUCN 2025)

##### Distribution

Vietnam (entire country: Nguyen et al. (2009)), Bangladesh, China, Myanmar, Cambodia, Laos, Thailand and Malaysia (Nguyen et al. 2009).

##### Notes

Previously recorded from BVNP by Nguyen et al. (2009), not observed during the present surveys.

#### Hebius boulengeri

(Gressitt,1937)

D8531F25-7CED-561C-8D35-571BDD4C2FA1

##### Materials

- **Type status:** Other material. **Occurrence:** catalogNumber: VNUF R.2019.03; individualCount: 1; sex: female; lifeStage: adul; occurrenceID: 4938FBCD-591A-5519-ABCA-FA529CB4AD05; **Taxon:** genus: Hebius; specificEpithet: *boulengeri*; **Location:** country: Vietnam; stateProvince: Hanoi; municipality: Ba Vi; locality: Ba Vi National Park; verbatimElevation: 805 m; verbatimCoordinates: 21 04.409N, 105 21.902E; verbatimCoordinateSystem: degrees decimal minutes; **Event:** eventDate: 6 June 2019; **Record Level:** institutionCode: VNUF

##### Native status

Not threatened

##### Conservation status

LC (IUCN 2025)

##### Distribution

In Vietnam, this species is known from Lao Cai and Ha Giang Provinces southwards to Dak Lak and Lam Dong Provinces. Elsewhere, from China, Laos, Cambodia and Thailand (Le et al. 2018). First record in BVNP.

##### Notes

The specimen was found in the evening on the road. The surrounding habitat was natural forest.

##### Diagnosis

Morphological characters of the specimens from BVNP agreed well with descriptions of Ziegler et al. (2007) and Le et al. (2018): SVL 410.0 mm, Tal 150.0 mm; head longer than wide (HL 17.3 mm, HW 9.87 mm), distinct from the neck; pupil round; loreal 1/1, not touching the eye; preocular 1/1; postoculars 1/1; anterior temporals 1/2, posterior temporals 2/2; supralabials 9/9, fourth to sixth in contact with the eye, 8^th^ largest; infralabials 10/10, first to seventh (both sides) bordering chin shields; dorsal scale rows 19–19–17, strongly keeled, except outer row smooth; ventrals 146; cloacal undivided; subcaudals 60, divided. Colouration in preservative: dorsal surface of body dark grey, dorsolateral stripe extends from the neck to the base of the tail. Posterior supralabials black with a median elongated cream blotch or streak, forming a postocular stripe extending on the neck; anterior supralabials white; first dorsal scale row smooth. The venter uniformly cream (Fig. 4).

#### Hebius chapaensis

(Bourret, 1934)

940DF012-EF5B-556F-83F5-A09BC781B26D

##### Native status

Not threatened

##### Conservation status

DD (IUCN 2025)

##### Distribution

Vietnam (BVNP: Luu et al. (2020)) and China (Luu et al. 2020).

##### Notes

Previously recorded from BVNP by Luu et al. (2020), observed during the present surveys.

#### Hebius khasiensis

(Boulenger, 1890)

99CB4624-9AE3-5DB0-BF2C-02FE95FF0067

##### Native status

Not threatened

##### Conservation status

LC (IUCN 2025)

##### Distribution

Vietnam (Ba Vi: Nguyen et al. (2009)), India, China, Myanmar, Laos, and Cambodia (Nguyen et al. 2009).

##### Notes

Previously recorded from BVNP by Nguyen et al. (2009), observed during the present surveys.

#### Hebius sauteri

(Boulenger, 1909)

7ABE5377-8E24-5407-ADE3-FBB2B7056193

##### Native status

Not threatened

##### Conservation status

LC (IUCN 2025)

##### Distribution

Vietnam (Ba Vi: Nguyen et al. (2009)), China, Taiwan and Laos (Nguyen et al. 2009).

##### Notes

Previously recorded from BVNP by Nguyen et al. (2009), not observed during the present surveys.

#### Opisthotropis lateralis

Boulenger, 1903

134E3A28-C368-5A98-8875-630A021400FF

##### Native status

Not threatened

##### Conservation status

LC (IUCN 2025)

##### Distribution

Vietnam (BVNP: Luu et al. (2020)) and China (Luu et al. 2020).

##### Notes

Previously recorded from BVNP by Luu et al. (2020), observed during the present surveys.

#### Rhabdophis callichroma

(Bourret, 1934)

19EF44A5-C36A-587B-A764-AF1092EA4CF5

##### Native status

Not threatened

##### Conservation status

DD (IUCN 2025)

##### Distribution

Vietnam (Ba Vi: Nguyen et al. (2009)) and China (Nguyen et al. 2009).

##### Notes

Previously recorded from BVNP by Nguyen et al. (2009), not observed during the present surveys.

#### Rhabdophis helleri

(Schmidt, 1925)

D8F0866F-A484-5348-A318-05AB8DE94DE8

##### Native status

Not threatened

##### Conservation status

Not evaluated

##### Distribution

Vietnam (Ba Vi: Nguyen et al. (2009)), Indian, Nepal, Bangladesh, China and Myanmar (David and Vogel 2021).

##### Notes

Previously recorded from BVNP by Nguyen et al. (2009), observed during the present surveys.

#### Trimerodytes percarinatus

(Boulenger, 1899)

E79EFAF2-A796-5214-A1DE-888314B79623

##### Materials

- **Type status:** Other material. **Occurrence:** catalogNumber: VNUF R.2024.13; individualCount: 1; sex: female; lifeStage: adult; occurrenceID: D9F5ADF4-F495-5D2A-B2E7-DCACABDA10D2; **Taxon:** genus: Trimerodytes; specificEpithet: *percarinatus*; **Location:** country: Vietnam; stateProvince: Hanoi; municipality: Ba Vi; locality: Ba Vi National Park; verbatimElevation: 700 m; verbatimCoordinates: 21 04.569N, 105 22.063E; verbatimCoordinateSystem: degrees decimal minutes; **Event:** eventDate: 10 July 2024; **Record Level:** institutionCode: VNUF

##### Native status

Not threatened

##### Conservation status

LC (IUCN 2025)

##### Distribution

In Vietnam, this species has previously been reported from Lao Cai Province in the north, southwards to Dong Nai Province. Elsewhere, this species has been reported from India, China, Taiwan, Myanmar, Laos and Thailand (Nguyen et al. 2009). First record in BVNP.

##### Notes

The specimen was found in the early morning at 1:55 h under the stream in French Camping area. The surrounding habitat were secondary forest, streams, bamboo forest and banana forest. The relative temperature was 25°C and the humidity was 88%.

##### Diagnosis

Morphological characters of the specimens from BVNP agreed well with descriptions of Hecht et al. (2013) and Le et al. (2018): SVL 750.0 mm, Tal 265.0 mm; head longer than wide (HL 30.0 mm, HW 18.0 mm), distinct from the neck; pupil round; loreal 1/1, not touching the eye; preocular 1/1; postoculars 3/3; anterior temporals 2/2, posterior temporals 2/2; supralabials 9/9, fourth to fifth in contact with the eye, 7^th^ largest; infralabials 10/10, first to fìfth bordering chin shields; dorsal scale rows 19–19–17, keeled; except the outer row; ventrals 140; cloacal divided; subcaudals 79, divided. Colouration in life: dorsal head olive-grey, brown body with 32 Y-shape bands on back, ventral yellowish-cream anteriorly and turning into dark grey posteriorly (Fig. 4).

#### Pareatidae

15F0AAA8-A549-5B77-AD31-593F586E3251

#### Pareas hamptoni

(Boulenger, 1905)

B90CDC01-891A-5FC5-B72D-E56DA6934143

##### Native status

Not threatened

##### Conservation status

LC (IUCN 2025)

##### Distribution

Vietnam (Ba Vi: Luu et al. (2020)), China, Myanmar, Laos and Cambodia (Luu et al. 2020).

##### Notes

Previously recorded from BVNP by Luu et al. (2020), observed during the present surveys.

#### Pareas margaritophorus

(Jan, 1866)

7D7C655C-1B86-5C5E-BBB0-556320ECCB28

##### Native status

Not threatened

##### Conservation status

LC (IUCN 2025)

##### Distribution

Vietnam (Ba Vi: Nguyen et al. (2009)), China, Myanmar, Laos, Cambodia, Thailand and Malaysia (Nguyen et al. 2009).

##### Notes

Previously recorded from BVNP by Nguyen et al. (2009), observed during the present surveys.

#### Pseudoxenodontidae

9305795B-F9DE-5A2D-8226-814B13EC1C27

#### Pseudoxenodon bambusicola

Vogt, 1922

4932071B-D29A-548A-8B6B-70E6010D0E39

##### Native status

Not threatened

##### Conservation status

LC (IUCN 2025)

##### Distribution

Vietnam (Ba Vi: Nguyen et al. (2009)), China and Laos (Nguyen et al. 2009).

##### Notes

Previously recorded from BVNP by Nguyen et al. (2009), observed during the present surveys.

#### Xenodermidae

B29F6F09-DDD2-5474-86D4-5A5796427CF4

#### Achalinus cf. juliani

Ziegler, Nguyen, Pham, Nguyen, Pham, Van Schingen, Nguyen & Le, 2019

6401677B-CBAD-57FD-BAD2-19509A1FEAAC

##### Materials

- **Type status:** Other material. **Occurrence:** catalogNumber: IB A.2025.099; individualCount: 1; sex: male; lifeStage: adult; occurrenceID: 6F11F8B3-2021-5F0A-B380-24A4431A9525; **Taxon:** genus: Achalinus; specificEpithet: cf. *juliani*; **Location:** country: Vietnam; stateProvince: Hanoi; municipality: Ba Vi; locality: Ba Vi National Park; verbatimElevation: 900 m; verbatimCoordinates: 21 04.331N, 105 21.752E; verbatimCoordinateSystem: degrees decimal minutes; **Event:** eventDate: 20 July 2025; **Record Level:** institutionCode: IB

##### Native status

Not threatened

##### Conservation status

Not evaluated

##### Distribution

In Vietnam, this species only known from Quang Ninh and Bac Giang Provinces. Elsewhere, this species has been reported from China (Ziegler et al. 2019). First record in BVNP.

##### Notes

The specimen was found on the main road at 22:00 h. The surrounding habitat was evergreen forest. The relative temperature was 25°C and the humidity was 90%.

##### Diagnosis

Morphological characters of the specimens from BVNP almost agreed with descriptions of Ziegler et al. (2019): SVL 305.0 mm, Tal 90.0 mm; head longer than wide (HL 13.6 mm, HW 6.0 mm), distinct from the neck; pupil round; loreal 1/1, not touching the eye; anterior temporals 2/2, posterior temporals 2/2; supralabials 6/6, fourth to fifth in contact with the eye, 6^th^ largest; infralabials 6/6, dorsal scale rows 25–23–23, keeled; ventrals 150; cloacal divided; subcaudals 64, divided. Colouration in life: dorsum is yellowish-brown with a dark greyish-brown longitudinal mid-dorsal stripe on the body, the dorsal tail surface is cream (Fig. 4).

#### Elapidae

95BEC028-5E74-5C6C-A1CF-A03CBF61D580

#### Bungarus fasciatus

(Schlegel, 1801)

1181ED8E-3A0B-5C20-8AC5-34D421DECEC4

##### Native status

NT (VNREDLIST 2024)

##### Conservation status

LC (IUCN 2025)

##### Distribution

Vietnam (Ba Vi: Nguyen et al. (2009)), Bangladesh, Burma, Cambodia, China, India, Bhutan, Nepal, Indonesia, Laos, Malaysia, Singapore and Thailand (Nguyen et al. 2009).

##### Notes

Previously recorded from BVNP by Nguyen et al. (2009), observed during the present surveys.

#### Naja atra

Cantor, 1842

ECBBC950-B41E-5555-AE63-C3FD778727FB

##### Native status

VU (VNREDLIST 2024)

##### Conservation status

VU (IUCN 2025)

##### Distribution

Vietnam (Ba Vi: Nguyen et al. (2009)), China, Taiwan and Laos (Nguyen et al. 2009).

##### Notes

Previously recorded from BVNP by Nguyen et al. (2009), not observed during the present surveys.

#### Ophiophagus hannah

(Cantor, 1836)

6F8C8B64-EB40-5DBE-9A97-3ADCCC665BFA

##### Native status

CR (VNREDLIST 2024)

##### Conservation status

VU (IUCN 2025)

##### Distribution

The geographical distribution of the nominate species, as restricted here, extends from extreme eastern Pakistan, across the sub-Himalayan Region of Kashmir, northern India, Nepal, Bhutan, Tibet and south to the Godavari-Mahanadi-Ganges deltas of the Circar Coast in eastern India, east to the eastern coast of China, including Hong Kong, the range extending south to Indo-China, including Myanmar, Laos, Vietnam (Ba Vi: Nguyen et al. (2009)), Cambodia and parts of Thailand, presumably north of the Isthmus of Kra (Das et al. 2024).

##### Notes

Previously recorded from BVNP by Nguyen et al. (2009), observed during the present surveys.

#### Sinomicrurus macclellandi

(Reinhardt, 1844)

38DC8276-7B02-5909-B403-C87109647C67

##### Native status

Not threatened

##### Conservation status

LC (IUCN 2025)

##### Distribution

Vietnam (Ba Vi: Nguyen et al. (2009)), India, Nepal, Taiwan, Thailand, Myanmar, China, Laos and Japan (Nguyen et al. 2009).

##### Notes

Previously recorded from BVNP by Nguyen et al. (2009), observed during the present surveys.

#### Viperidae

651B77F0-7D9E-5F07-B84F-B1B0AA3D1C53

#### Ovophis monticola

(Günther, 1864)

5278FCE8-8A50-5CCB-BFD1-6715E1DFECEE

##### Native status

Not threatened

##### Conservation status

LC (IUCN 2025)

##### Distribution

Vietnam (Ba Vi: Nguyen et al. (2009)), India, Nepal, Taiwan, Thailand, Myanmar, China, Laos, Cambodia, Indonesia and Malaysia (Nguyen et al. 2009).

##### Notes

Previously recorded from BVNP by Nguyen et al. (2009), not observed during the present surveys.

#### Protobothrops mucrosquamatus

(Cantor, 1839)

0C95DD7C-4AA1-5284-A55F-B6A08EE8FAD3

##### Native status

Not threatened

##### Conservation status

LC (IUCN 2025)

##### Distribution

Vietnam (Ba Vi: Nguyen et al. (2009)), India, Bangladesh, China and Myanmar (Nguyen et al. 2009).

##### Notes

Previously recorded from BVNP by Nguyen et al. (2009), observed during the present surveys.

#### Trimeresurus albolabris

Gray, 1842

510800CB-11DC-510A-AA18-582DFFD72E07

##### Native status

Not threatened

##### Conservation status

LC (IUCN 2025)

##### Distribution

Vietnam (Ba Vi: Nguyen et al. (2009)), India, China, Myanmar, Laos, Thailand and Cambodia (Nguyen et al. 2009).

##### Notes

Previously recorded from BVNP by Nguyen et al. (2009), observed during the present surveys.

#### Trimeresurus stejnegeri

Schmidt, 1925

0DCB49C3-0005-5049-AD3B-F15F24E967C9

##### Materials

- **Type status:** Other material. **Occurrence:** catalogNumber: IB A.2025.066; individualCount: 1; sex: female; lifeStage: adult; occurrenceID: BD88DCBF-44F1-59A8-B230-0F718EC9547C; **Taxon:** genus: Trimeresurus; specificEpithet: *stejnegeri*; **Location:** country: Vietnam; stateProvince: Hanoi; municipality: Ba Vi; locality: Ba Vi National Park; verbatimElevation: 1000 m; verbatimCoordinates: 21 03.658N, 105 21.737E; verbatimCoordinateSystem: degrees decimal minutes; **Event:** eventDate: 27 June 2025; **Record Level:** institutionCode: IB

##### Native status

Not threatened

##### Conservation status

LC (IUCN 2025)

##### Distribution

In Vietnam, this species has previously been reported from Lao Cai to Hoa Binh Provinces in the north, southwards to Dong Nai Province. Elsewhere, this species has been reported from China, Taiwan and Myanmar (Nguyen et al. 2009). First record in BVNP.

##### Notes

The specimen was found on tree leaves at 23:30 h. The surrounding habitat was evergreen forest. The relative temperature was 25°C and the humidity was 89%.

##### Diagnosis

Morphological characters of the specimens from BVNP agreed well with descriptions of Smith (1943): SVL 786.0 mm, TaL 122.0 mm; head longer than wide (HL 23.36 mm, HW 16.73 mm), distinct from neck; pupil vertically elliptical; loreal 1/1; preocular 3/3; postoculars 3/3; anterior temporals 3/3, posterior temporals 2/2; supralabials 10/10, fourth to sixth in contact with the eye, 7^th^ largest; infralabials 10/11, first to fifth bordering chin shields; dorsal scale rows 19–19–15, keel; ventrals 155; cloacal entire; subcaudals 69, divided. Colouration in life: dorsal surfaces are green, ventral surfaces are pale green. A light-coloured stripe extending along the flanks and base of the tail, bordered ventrally by red, restricted to the first scale row. The tail tip is red-brown (Fig. 4).

#### Geoemydidae

2B1FE20E-7EC9-541B-B84B-CAF48561C196

#### Cuora mouhotii

(Gray, 1862)

3E84D3B0-0F56-55A9-87F3-424076447F78

##### Native status

EN (VNREDLIST 2024)

##### Conservation status

EN (IUCN 2025)

##### Distribution

Vietnam (Ba Vi: Nguyen et al. (2009)), India, China, Myanmar, Laos and Thailand (Nguyen et al. 2009).

##### Notes

Previously recorded from BVNP by Nguyen et al. (2009), not observed during the present surveys.

#### Mauremys sinensis

(Gray, 1834)

A4338D07-9BD8-59A9-813F-EEAF89D312B3

##### Native status

CR (VNREDLIST 2024)

##### Conservation status

CR (IUCN 2025)

##### Distribution

Vietnam (Ba Vi: Nguyen et al. (2009)), China and Taiwan (Nguyen et al. 2009).

##### Notes

Previously recorded from BVNP by Nguyen et al. (2009), not observed during the present surveys.

#### Testudinidae

B5E181A7-9911-5467-915F-B6DF9E1FA377

#### Indotestudo elongata

(Blyth, 1854)

8582E063-0CDC-5A62-BAD8-A3CAE09979B5

##### Native status

CR (VNREDLIST 2024)

##### Conservation status

CR (IUCN 2025)

##### Distribution

Vietnam (Ba Vi: Nguyen et al. (2009)), India, Nepal, Bangladesh, China, Myanmar, Laos, Thailand, Cambodia and Malaysia (Nguyen et al. 2009).

##### Notes

Previously recorded from BVNP by Nguyen et al. (2009), not observed during the present surveys.

#### Trionychidae

EC0AF58F-89AF-5E6D-BE12-60A214E6FFFB

#### Pelodiscus variegatus

Farkas, Ziegler, Pham, Ong & Fritz, 2019

3B83A6B6-9756-5BBF-BFEF-28889711241F

##### Native status

Not evaluated

##### Conservation status

VU (IUCN 2025)

##### Distribution

Vietnam (Bac Giang, Tuyen Quang, Yen Bai, Ba Vi (Nguyen et al. 2009), Phu Tho, Hai Duong, Hai Hung, Ninh Binh, Ha Tinh, Quang Nam and Quang Binh Provinces) and China (Farkas et al. 2019).

##### Notes

Previously recorded from BVNP by Nguyen et al. (2009), not observed during the present surveys.

## Discussion

**Species richness**

**Figure 5.**
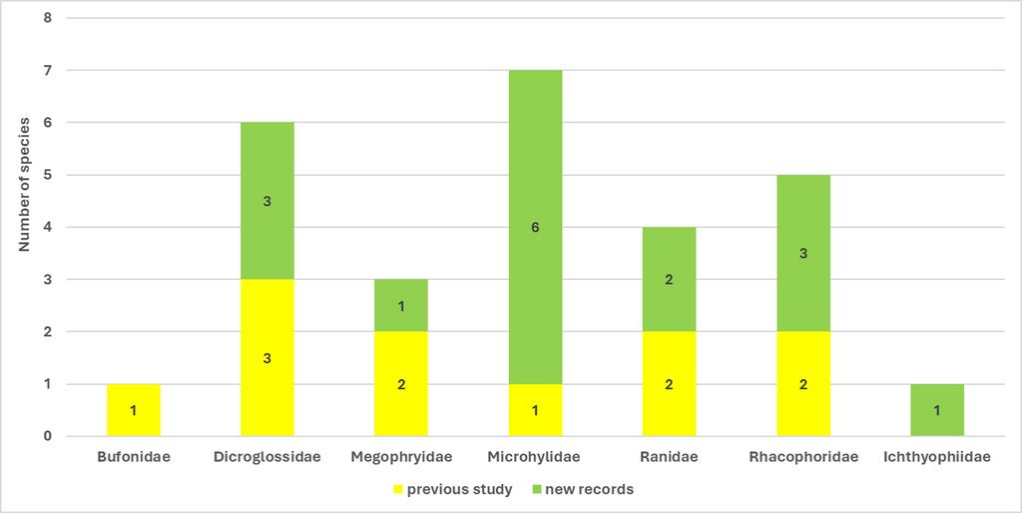
Species richness of amphibian families from BVNP.

**Figure 6.**
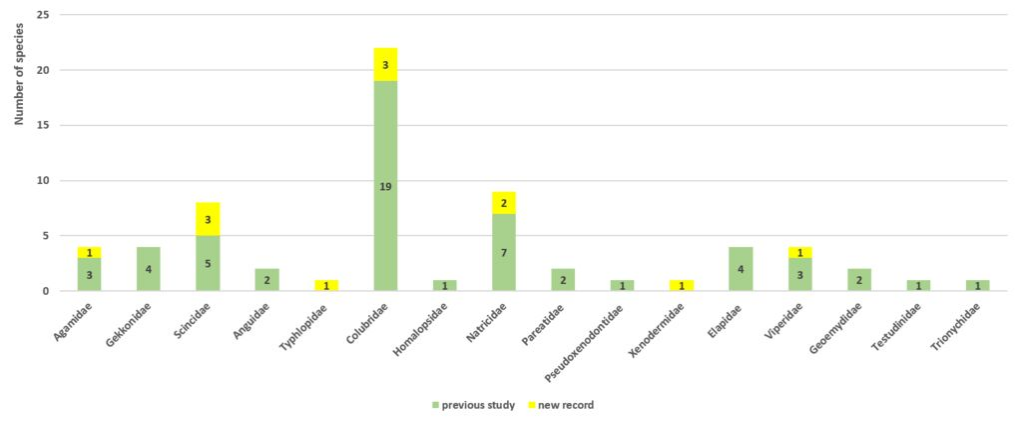
Species richness of reptile families from BVNP.

All 28 species are reported first time from BVNP, although some species are quite common and widespread in Vietnam (*Occidozyga martensii*, *Microhyla pulchra*, *Sphenomorphus indicus*) (Nguyen et al. 2009, Hecht et al. 2013, Le et al. 2021). This means that the herpetofaunal inventory is still not sufficient in BVNP. Even if the inventory is not complete, our present study clearly demonstrates how BVNP houses high species richness. Combining with the previous data from Nguyen et al. (2009), Nguyen et al. (2011b), Chen et al. (2018), Luu et al. (2020), Orlov et al. (2020), Nguyen et al. (2021) and Pham et al. (2025), our study makes the total species number in BVNP up to 94 including 27 species of amphibians and 67 species of reptiles (30% of the species were added by this study). In the amphibian component (Fig. 5), the most diversified family is Microhylidae with seven species (six new records, approximately 26% from total species of amphibians). Next is Dicroglossidae, Rhacophoridae, Ranidae and Megophryidae with six species (three new records, 22.2%), five species (three new records, 18.5%), four species (two new records, 14.8%) and three species (one new record, 11.1%), respectively. Bufonidae and Ichthyophiidae only have one species (3.7%), but Ichthyophiidae is the new family record from BVNP. As Microhylidae is a large group and its taxonomy has been greatly changed even recently, we could expect similar new findings in the family from poorly-surveyed areas in the NPs. In the reptile component (Fig. 6), the most diversified family is Colubridae with 22 species (three new records, approximately 32.8% from total species of reptiles). Next are Natricidae and Scincidae with nine species (two new records, 13.43%) and eight species (three new records, 12.0%). Agamidae and Viperidae, each with four species (one new record, 6.0% for each family), Elapidae and Gekkonidae each with four species (no new records, 6.0%). The rest of the family only have one (1.5%) and two (3.0%) species, but Typhlopidae and Xenodermidae are two new records as families in this area. Like the case in amphibians, we could expect similar and more findings in Colubridae (large group with many recent taxonomic changes) and Typhlopidae and Xenodermidae (small group, but with less known ecology and distribution) in future.

**Taxonomic changes in species recorded from BVNP**

At present, a total of 27 species of amphibians belonging to 20 genera (six families, two orders) and 67 species of reptiles belonging to 44 genera (16 families, two orders) are known from BVNP. It is noted that the taxonomic assignments of some species have been changed. For example, *Limnonectes kuhlii* (Tschudi, 1838) was changed to *L. bananensis* Ye, Fei, Xie & Jiang, 2007 by McLeod (2010). *Xenophrys major* has restricted distribution in the northeast region of India (Mahony et al. 2018) and previous records of the species in Vietnam was changed to be the *X. maosonensis* species complex, based on molecular evidence. *Gonyosoma prasinum* (Blyth, 1854) from BVNP was changed to *G. coeruleum* Liu, Hou, Ye Htet Lwin, Wang & Rao, 2021 by David et al. (2022). *Rhabdophis subminiatus* (Schlegel, 1837) from BVNP was changed to *R. helleri* by David and Vogel (2021). *Ichthyophis bannanicus* Yang, 1984 in Vietnam was synonymised with *Ichthyophis kohtaoensis* Taylor, 1960 by Nishikawa et al. (2021). *Lycodon meridionalis* (Bourret, 1935) has been changed to *L. flavozonatus* Pope (1928) (Li et al. 2025) and *Hypsiscopus plumbea* (Boie, 1827) to *H. murphyi* Bernstein, Voris, Stuart, Phimmachak, Seateun, Sivongxay, Neang, Karns, Andrews, Osterhage, Phipps & Ruane, 2022 (Bernstein et al. 2022). *Xenochrophis flavipunctatus* (Hallowell, 1860) from BVNP was changed to *Fowlea flavipunctata* (Hallowell, 1860) by Amarasinghe et al. (2022). *Polypedates leucomystax* (Gravenhorst, 1829) from Vietnam was separated into *P. mutus* (Smith, 1940) and *P. megacephalus* Hallowell, 1861 by Kuraishi et al. (2012), although *P. leucomystax* is still distributed throughout Vietnam according to Nguyen et al. (2009). *Lycodon subcinctus* Boie, 1827 from BVNP was changed to *L. neomaculatus* Nguyen, Lee, Pauwels, Kennedy-Gold, Poyarkov, David & Vogel, 2024 by Nguyen et al. (2024). *Pelodiscus sinensis* Wiegmann, 1835 from BVNP was changed to *P. variegatus* Farkas, Ziegler, Pham, Ong & Fritz, 2019 by Farkas et al. (2019).

**Survey design, observed species and temporal limitations**

Field surveys conducted between April and August from 2019 to 2025 documented a total of 94 amphibian and reptile species in BVNP, including 28 species representing new distributional records for the Park. In addition, 41 previously recorded species were re-observed during the present surveys, most of which were encountered along forest roads and trails, highlighting the importance of roadside habitats for detectability in this protected area. Several semi-aquatic species, particularly within Natricidae (e.g. *Hebius chapaensis*, *H. khasiensis*, *Opisthotropis lateralis*), were primarily observed in close proximity to streams and wet habitats. Road mortality was frequently recorded, especially for snakes, reflecting the high level of tourism and vehicle traffic within the Park; dead individuals were commonly encountered on roads, notably *Rhabdophis helleri* and *Dopasia ludovic*. In addition, several anuran species were often observed moving on to roads following rainfall events. The remaining 25 species reported in earlier studies were not detected during the present surveys, likely due to the restricted temporal scope of sampling, which was conducted exclusively at night and did not include standardised daytime surveys or broader seasonal coverage. As a result, species with predominantly diurnal, fossorial, cryptic or highly seasonal activity patterns may have been under-detected. Although no statistical analyses of diurnal or seasonal variation were performed, the targeted nocturnal survey design is appropriate for maximising detectability of tropical herpetofauna and effectively fulfils the primary objectives of documenting species composition and new distributional records. Future studies incorporating daytime surveys, together with pitfall, funnel or bottle trapping, would be valuable for re-detecting diurnal or rarely encountered species, such as *Plestiodon tamdaoensis* and *Scincella devorator*, as well as for improving detection of rare *Rhabdophis callichroma*, *Goniurosaurus lichtenfelderi* or well-camouflaged taxa freshwater turtles and potentially even salamander lineages that may occur in BVNP, but remain undocumented.

**Estimation of complete species richness**

**Figure 7.**
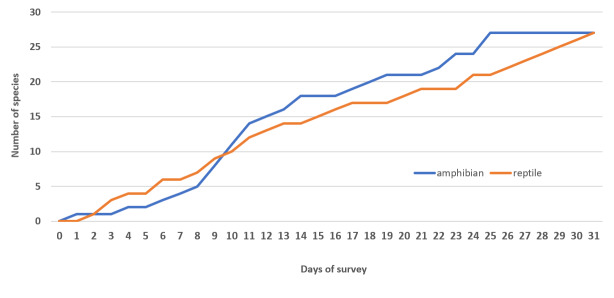
Species increment curves.

During 31 days of field surveys, a total of 100 individuals from 54 species were recorded. Amongst them, new records of amphibian and reptile species were obtained in 18 days. In the first 10 days, the number of species in both groups increased sharply up to 10 species. In the following 10 days, the number of amphibian species continued to rise constantly, reaching 20 species, while reptile species only increased by eight. During the final 11 days, the number of reptile species surged from 18 to 27, while amphibians increased modestly from 21 to 27 species. Although the increment trends are slightly different between amphibians and reptile, the both increment curves do not come to any asymptotic lines (Fig. 7). Thus, it would be safe to say that the number of amphibian and reptile species will increase more in future.

**Species richness and sampling completeness**

Sampling completeness for amphibians and reptiles in BVNP was assessed using sample-based rarefaction curves and incidence-based richness estimators implemented in R with the vegan package (Oksanen et al. 2022), following the framework of Mao Tau rarefaction and non-parametric estimators proposed by Colwell et al. (2004). In this approach, Mao Tau rarefaction represents the expected cumulative number of species as a function of sampling effort, here expressed as the number of survey days, based on repeated random re-ordering of samples.

**Figure 8.**
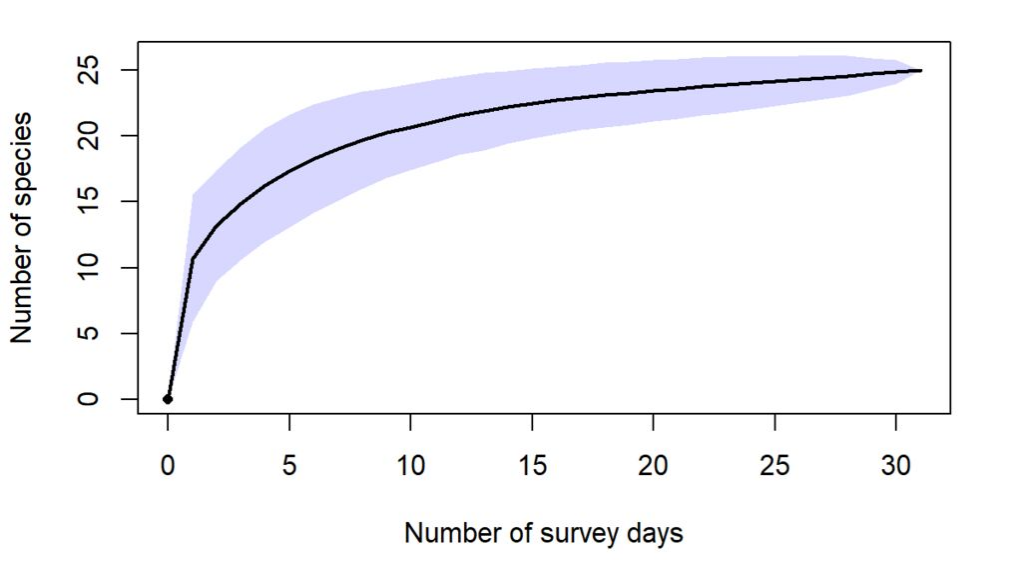
Rarefaction curve of amphibian species, based on presence–absence data.

For amphibians, 25 species were recorded during 31 survey days (Observed S = 25). The rarefaction curve (Fig. 8) shows a rapid initial increase followed by a slower accumulation phase, but does not reach a clear asymptote. Incidence-based richness estimators predict slightly higher diversity than observed, with estimated values ranging from 26.6 species (Bootstrap) to 32.6 species (Jackknife 2) and Chao2 = 30.8, suggesting that most amphibian species present were detected, while only a small number may remain undetected. The non-detection of two previously reported but widespread species (*Hoplobatrachus chinensis* and *Hylarana taipehensis*) is likely related to habitat preference, as these taxa are mainly associated with agricultural landscapes, wetlands and non-forested habitats, rather than forest environments targeted during the present surveys.

**Figure 9.**
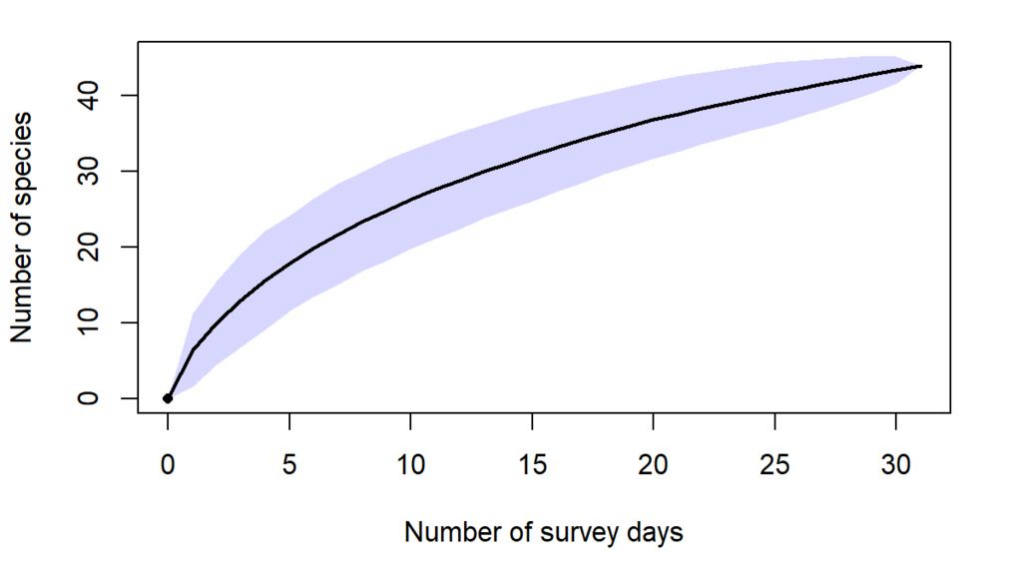
Rarefaction curve of reptile species, based on presence–absence data.

For reptiles, although 67 species are known from the combined checklist, only 44 species were observed during 31 survey days (Observed S = 44). The rarefaction curve (Fig. 9) increases more gradually and does not reach a clear asymptote and richness estimators indicate substantially higher expected diversity, ranging from 51.6 species (Bootstrap) to 72.8 species (Jackknife 2), with Chao2 = 70.1. The absence of the remaining 23 previously reported species is most likely explained by differences in activity patterns and detectability rather than insufficient sampling effort, as the reptile assemblage includes diurnal taxa that are less likely to be encountered during nocturnal surveys, as well as nocturnal, but highly cryptic or fossorial species with low encounter rates or strong microhabitat specialisation. Despite these limitations, the current sampling effort is considered sufficient to document the majority of commonly encountered and regularly detectable reptile species and to support conclusions regarding new distributional records.

Overall, the present dataset greatly upgraded the herpetofaunal records in BVNP; meanwhile, we need more exploration with additional and adjusted survey methods in order to encounter rare and diurnal species.

**Broader implications for Vietnam and Indo-Burma**

The present results demonstrate that, even in a relatively well-explored protected area such as BVNP, herpetofaunal diversity remains underestimated. The discovery of 28 new distributional records, together with the discrepancy between observed and estimated species richness, indicates that incomplete biodiversity knowledge is not limited to remote regions, but may also occur near major urban centres. Given the biogeographic importance of northern Vietnam, particularly in relation to the Red River system, updated local inventories contribute to a better understanding of regional biodiversity patterns in Vietnam and the Indo-Burma hotspot. In the context of ongoing habitat modification, tourism pressure and urban expansion, continued biodiversity assessments are essential for improving conservation planning both in BVNP and in other protected areas across Vietnam and Southeast Asia.
